# Supplementary material for: Novel FFPE proteomics method suggests prolactin induced protein as hormone induced cytoskeleton remodeling spatial biomarker
Source: Commun Biol. 2024 Jun 8;7:708. doi: 10.1038/s42003-024-06354-8 (PMC11162451; doi:10.1038/s42003-024-06354-8)
Supplement: Supplementary file 1 — Supplementary Information [file 42003_2024_6354_MOESM1_ESM.pdf]

**Novel FFPE Processing Method for Proteome Analysis Suggests Prolactin Induced Protein as Hormone Induced Cytoskeleton Remodeling Spatial Biomarker**

Jakub Faktor[*1], Sachin Kote[**1], Michal Bienkowski[2], Ted R. Hupp[1] and Natalia Marek-Trzonkowska[1]

[1]*International Centre for Cancer Vaccine Science, University of Gdansk, Kladki 24, 80-822, Gdansk, Poland*

[2]*Medical university of Gdansk, University of Gdansk, Mariana Smoluchowskiego 17, 80-214, Gdansk, Poland*

Jakub Faktor: jakub.faktor@ug.edu.pl; Sachin Kote: sachin.kote@ug.edu.pl

[*] Corresponding author jakub.faktor@ug.edu.pl

[**] Corresponding author sachin.kote@ug.edu.pl

# SUPPLEMENTARY INFORMATION

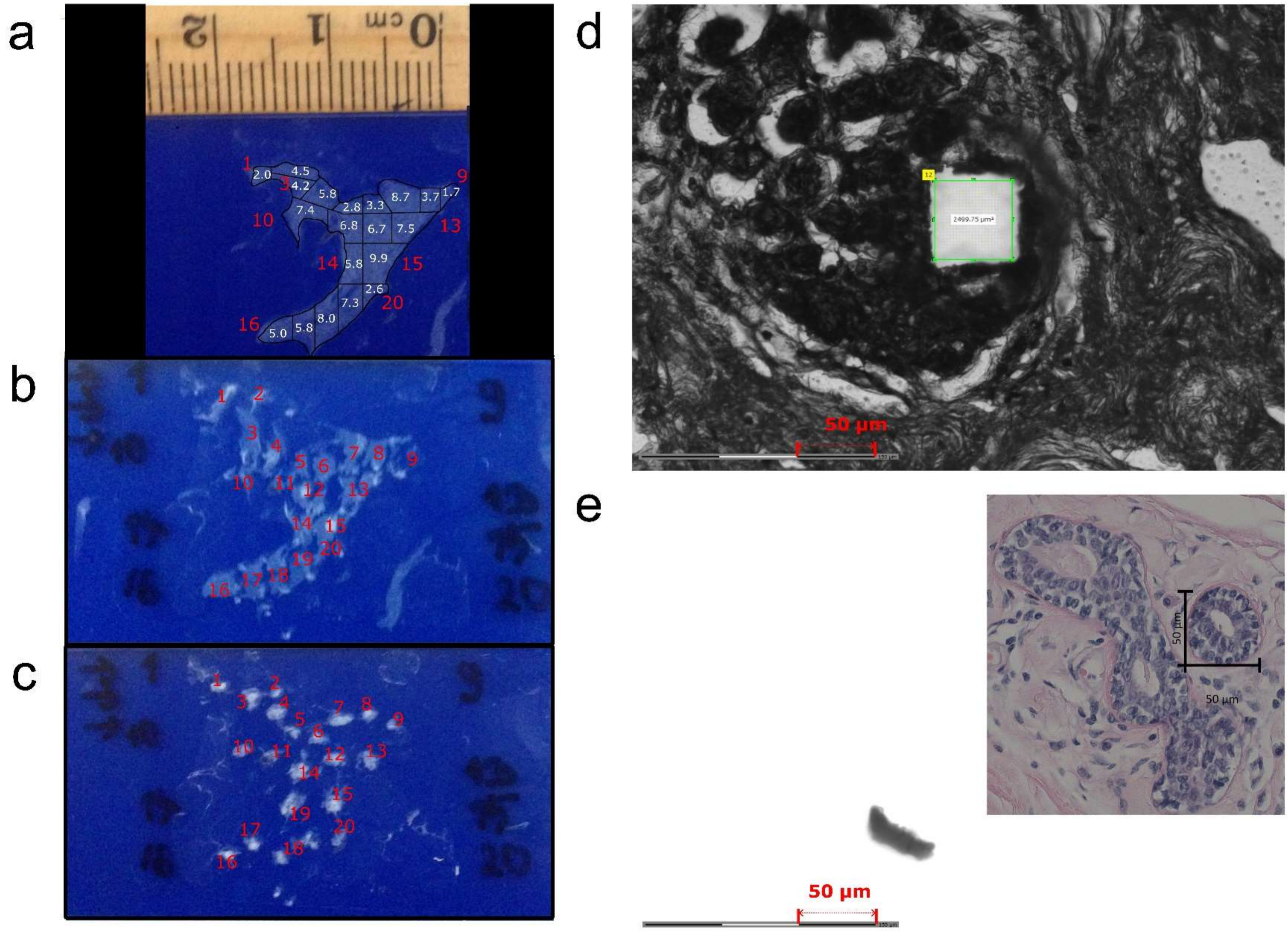


**Supplementary figure 1** Workflow of glass mounted BFPT macrodissection into 20 voxels by a razor and laser capture microdissection (LCM) of formalin fixed paraffin embedded (FFPE) breast glandular tissue voxel. a) An outline of 20 macrodissected FFPE voxels ranging from approx. 2 mm$^2$ up to 10 mm$^2$ area. White numbers depict area of voxel in mm$^2$ while red numbers show the assigned number of the voxel matching across the manuscript text. b) Separating individual voxels with a razor knife c) Scraping of FFPE tissue into stacks prior adding 3 µl of lysis buffer and transfer into a glass insert with a pipette. d) Hematoxylin stained 5 µm thick breast FFPE tissue slide after dissecting 50 µm lateral size FFPE voxel using LCM. e) LCM cut breast glandular FFPE tissue of 50 µm lateral size from Supplementary figure 1D after transferring into the adhesive cap of LCM tube and an example of a color image of hematoxylin stained mammary acini of 50 µm lateral size to estimate the cell count.

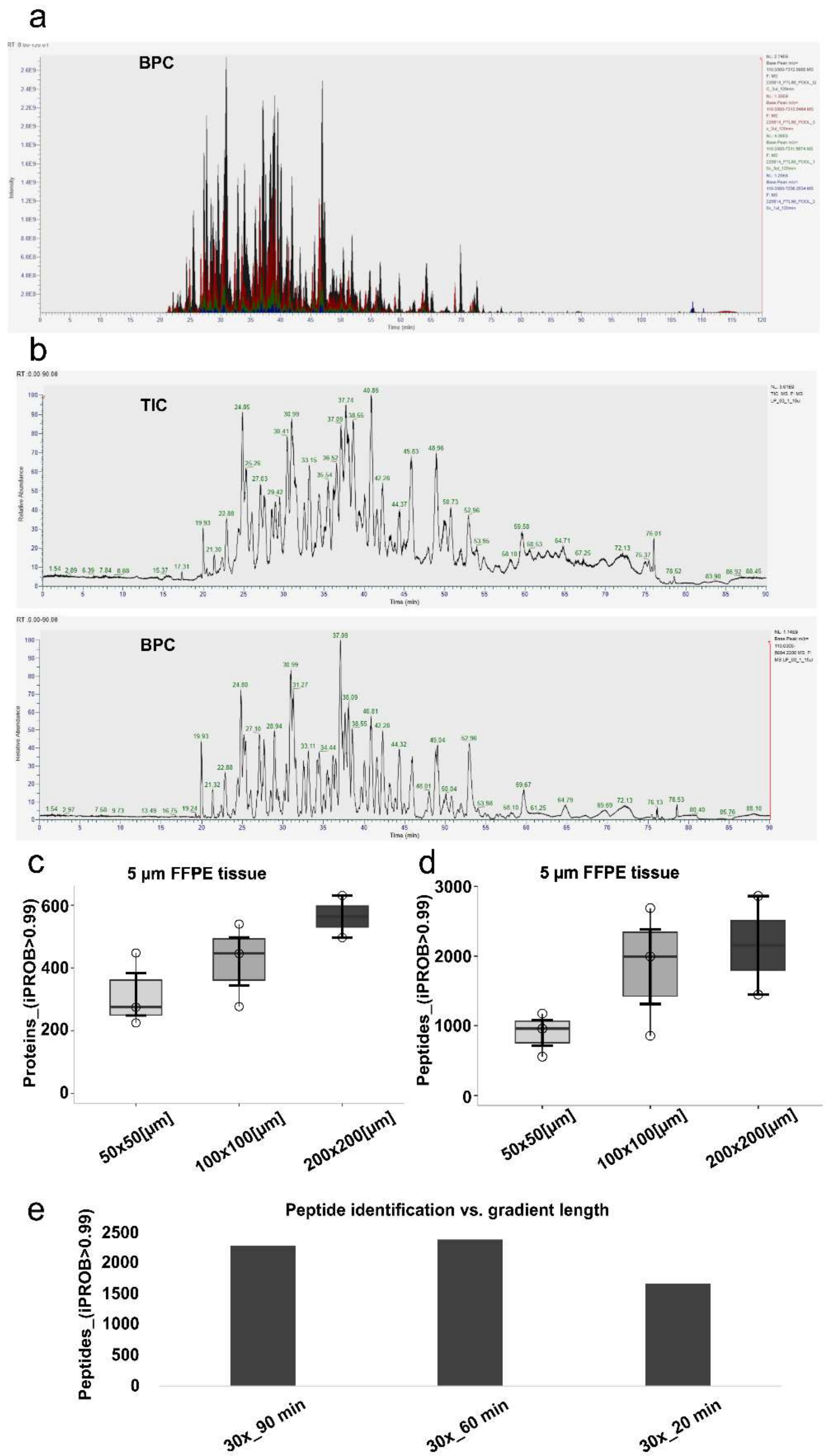


**Supplementary figure 2** Mass spectrometry quality control of In-insert processed BFPT samples and detection limit. a) Base peak chromatograms (BPC) at various dilutions of average macrodissected formalin fixed paraffin embedded breast tissue slide (BFPT) sample. The black color refers to BPC acquired from 1/10 of the total extracted material per average macrodissected BFPT voxel. The red color refers to BPC acquired from 1/30 of the total extracted material per an average macrodissected BFPT voxel. The green color refers to BPC acquired from 1/300 of the total extracted material per average macrodissected BFPT voxel and the blue color refers to BPC acquired from 1/1800 of the total extracted material per average macrodissected BFPT voxel. b) Total ion current chromatograms (TIC) and BPC referring to In-insert processed 50 µm lateral size LCM microdissected BFPT voxel. c) Number of protein group identifications in 50, 100 and 200 µm lateral size LCM microdissected hematoxylin stained BFPT voxels. d) Number of peptide identifications in 50, 100 and 200 µm lateral size LCM microdissected hematoxylin stained BFPT voxels. In c) and d) the error bars show standard error and the box extends from the lower to upper quartile, the line shows median. Actual values from the measurements are shown as individual circles. e) Testing the effect of gradient length on tryptic peptide identification on 30x diluted pool representing average macrodissected voxel. Gradients of 90, 60 and 20 min in length were tested. Gradients of 60 min does not show compromised tryptic peptide identification, which might be particularly important for high throughput In-insert spatial FFPE tissue analyses.

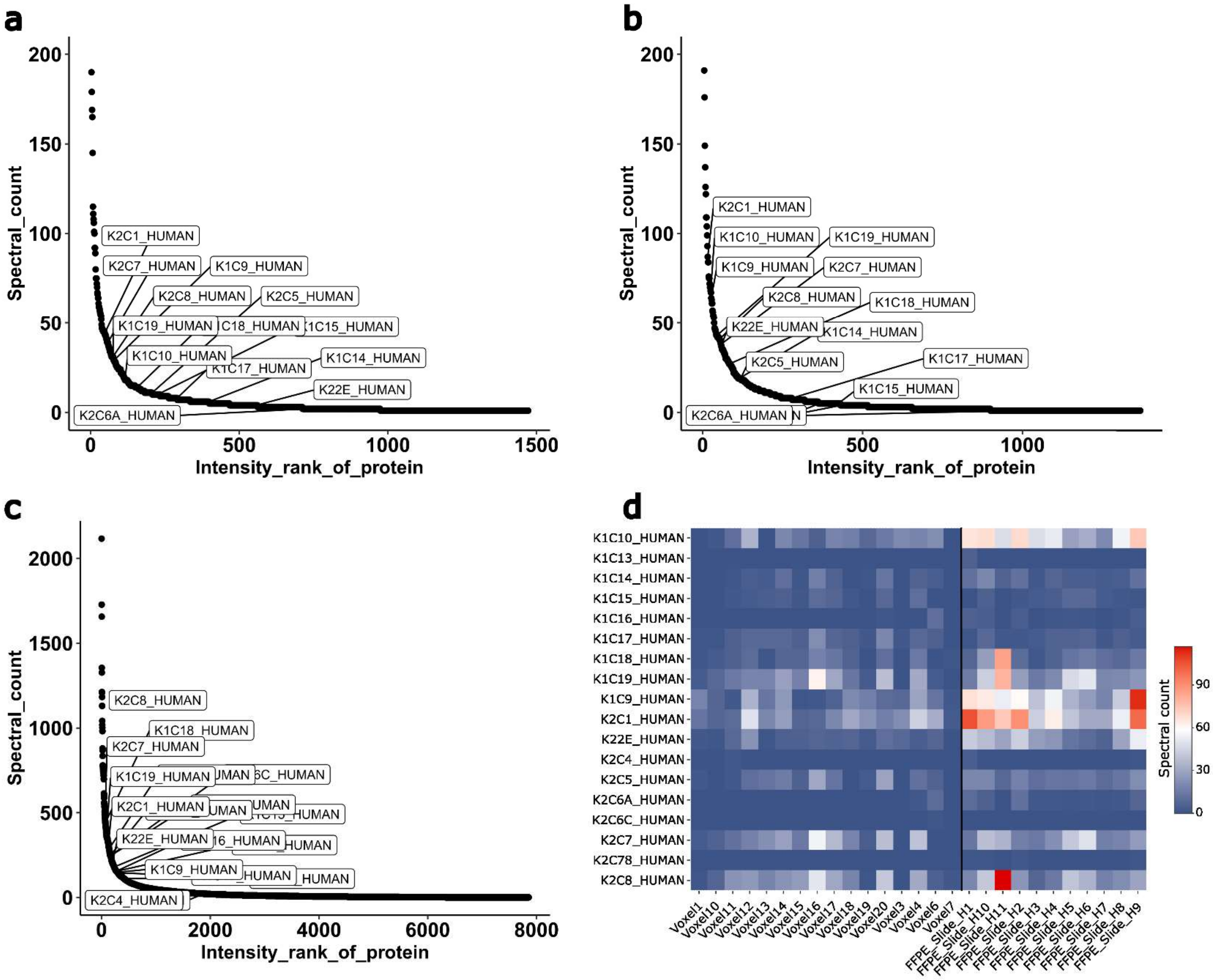

**Supplementary figure 3** Benchmarking keratin spectral counts from In-insert data to two independent mass spectrometry breast formalin fixed paraffin embedded tissue (FFPE) datasets. a) Illustrates the keratin spectral count in a formalin fixed paraffin embedded breast tissue (BFPT) voxels processed by In-insert method. b) Illustrates the keratin spectral count in BFPT slides processed by the protocol published by Weke et al. [1]. c) Shows total keratin spectral count in the publicly available dataset of FFPE breast cancer tissue labelled by tandem mass tag (TMT) approach [2]. d) A keratin spectral count heatmap comparing the spectral count of keratins in healthy BFPT slides from 6 donors, prepared by the method described by Weke et al. with the keratin spectral count in 17 BFPT voxels prepared using the In-insert method from a single donor's healthy BFPT slide [1]. Higher spectral count keratins are shown in shades of red, while low spectral count keratins are shown in shades of blue.

a

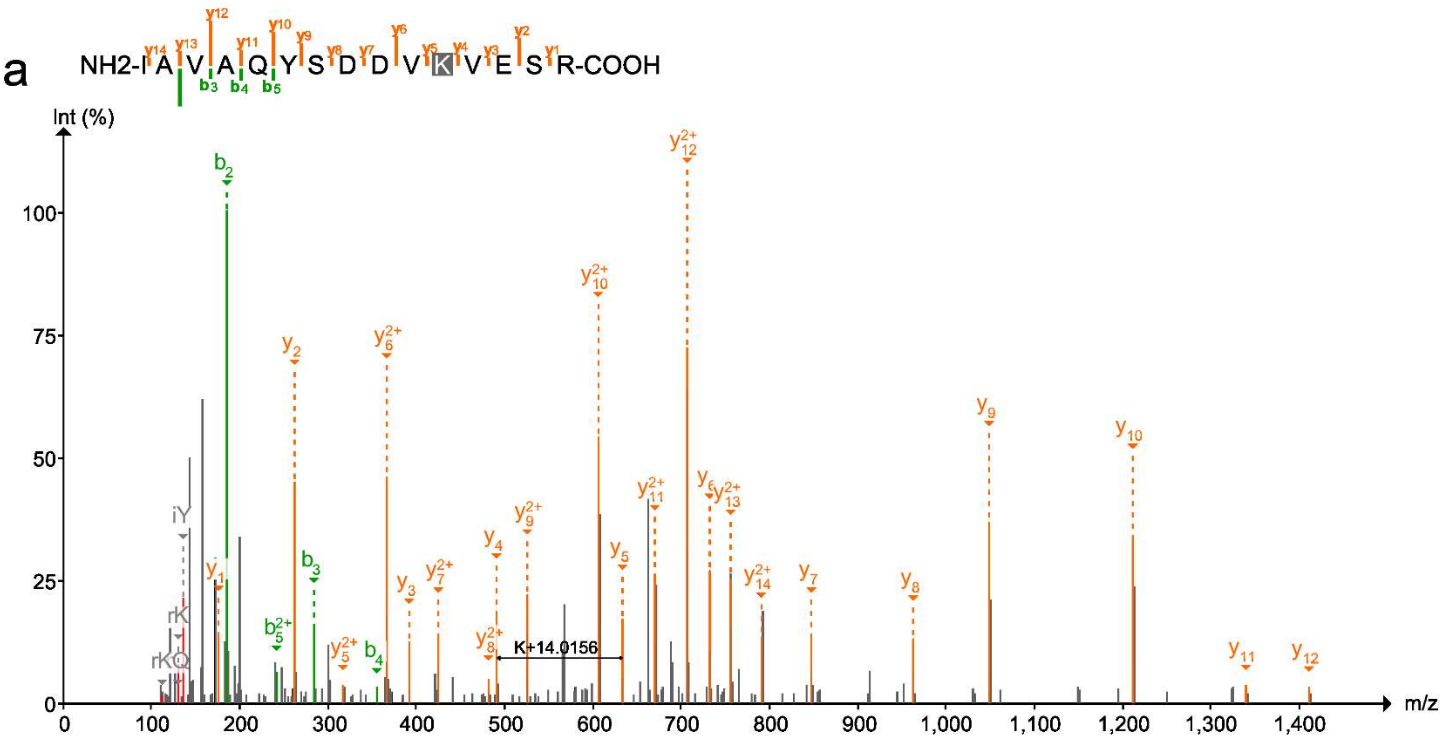


b

| Type | Index | AA | Original Mass | New Defined |
|---|---|---|---|---|
| Mod | 11 | K | +14.0156 | +14.0156 |

| | b | b++ | AA | y | y++ | |
|---|---|---|---|---|---|---|
| 1 | 114.0913 | 57.54931 | I | | | 15 |
| 2 | 185.1285 | 93.06787 | A | 1580.791 | **790.8994** | 14 |
| 3 | 284.1969 | 142.6021 | V | 1509.754 | **755.3808** | 13 |
| 4 | 355.234 | 178.1206 | A | **1410.686** | **705.8466** | 12 |
| 5 | 483.2926 | 242.1499 | Q | **1339.649** | **670.328** | 11 |
| 6 | 646.3559 | 323.6816 | Y | **1211.59** | **606.2987** | 10 |
| 7 | 733.3879 | 367.1976 | S | **1048.527** | **524.7671** | 9 |
| 8 | 848.4149 | 424.7111 | D | **961.4949** | **481.2511** | 8 |
| 9 | 963.4418 | 482.2245 | D | **846.4679** | **423.7376** | 7 |
| 10 | 1062.51 | 531.7587 | V | **731.441** | **366.2241** | 6 |
| 11 | 1204.621 | 602.814 | K | **632.3726** | **316.6899** | 5 |
| 12 | 1303.689 | 652.3482 | V | 490.262 | 245.6346 | 4 |
| 13 | 1432.732 | 716.8695 | E | 391.1936 | 196.1004 | 3 |
| 14 | 1519.764 | 760.3855 | S | 262.151 | 131.5791 | 2 |
| 15 | | | R | 175.119 | 88.06311 | 1 |

**Supplementary figure 4** Annotated spectrum and fragmentation evidence of a lysine methylated miscleaved peptide. a) The fragmentation spectrum of the NH2-IAVAQYSDDVK$_{\text{methylated}}$ (+14.0156 Da)VESR-COOH peptide shows the detection of y and b fragment ion series on Exploris480. The delta m/z between product ions y4 and y5 corresponds to a mass of 142.1106 Da, which represents mass of lysine minus the mass of water molecule plus mass of methyl modification (14.0156 Da). b) Fragmentation evidence of the NH2-IAVAQYSDDVK$_{\text{methylated}}$ (+14.0156 Da)VESR-COOH peptide is provided. Detected b fragment ions are highlighted in blue and y fragment ions are highlighted in orange. The bold fragment ions are proving the evidence of the methylated lysine in the peptide sequence.

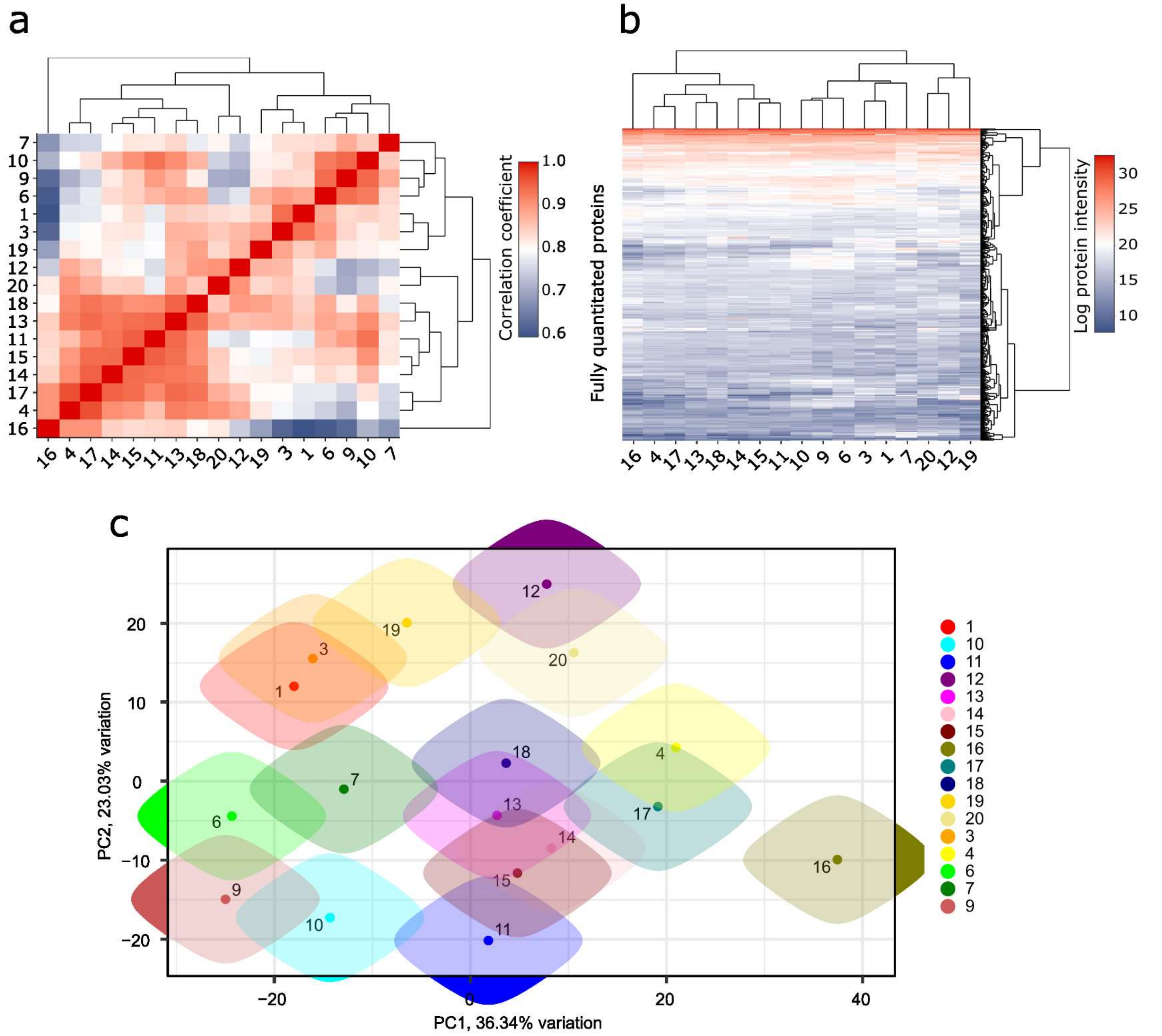


**Supplementary figure 5** Evaluation of data independent (DIA) quantitation accuracy and reproducibility within formalin fixed paraffin embedded breast tissue voxels subjected to In-insert method processing. a) Correlation heatmap illustrates favorable correlation coefficients for quantitative data, particularly among the neighboring voxels. Notably some voxels retrieved from edge of slide exhibit greater variation, displaying lower correlation coefficients compared to other voxels. b) Protein intensity heatmap demonstrates relatively similar patterns of quantitated protein intensities across voxels, further supporting observations from correlation heatmap. c) The Principal Analysis plot (PCA) plot highlights the clustering of voxels with similar protein intensity profiles, while voxels with distinct profiles are positioned farther apart, displaying higher proteotype dissimilarity.

| | log10 protein intensity (quantile normalized) | | | | | | | | | | | | | | | | |
|---|---|---|---|---|---|---|---|---|---|---|---|---|---|---|---|---|---|
| **Voxel** | **10** | **11** | **12** | **13** | **14** | **15** | **16** | **17** | **18** | **19** | **20** | **3** | **4** | **6** | **7** | **9** | **1** |
| PNPH_HUMAN | 16.43 | 12.21 | 9.79 | 14.11 | 10.44 | 10.02 | 11.36 | 9.94 | 9.87 | 9.14 | 13.00 | 16.82 | 15.54 | 10.48 | 13.13 | 19.66 | 13.84 |
| CAH1_HUMAN | 19.08 | 14.63 | 11.24 | 17.68 | 14.16 | 10.97 | 12.65 | 8.49 | 8.98 | 10.10 | 14.61 | 18.54 | 17.85 | 11.93 | 15.64 | 19.22 | 16.32 |
| ANGT_HUMAN | 15.87 | 18.62 | 16.68 | 11.32 | 15.44 | 15.60 | 16.07 | 8.62 | 13.33 | 14.49 | 10.30 | 11.65 | 14.54 | 11.42 | 18.30 | 16.30 | 18.79 |
| **B3AT_HUMAN** | 19.48 | 15.56 | 12.69 | 16.87 | 14.58 | 13.31 | 14.02 | 11.09 | 11.42 | 8.97 | 14.37 | 19.67 | 18.72 | 13.35 | 15.27 | 18.68 | 18.21 |
| CFAI_HUMAN | 14.29 | 16.76 | 14.69 | 11.21 | 13.45 | 13.20 | 13.48 | 10.77 | 10.91 | 13.58 | 12.56 | 11.01 | 12.93 | 9.23 | 17.60 | 14.91 | 17.63 |
| **GTR1_HUMAN** | 19.08 | 15.75 | 10.95 | 14.73 | 14.35 | 12.54 | 13.11 | 12.66 | 10.38 | 10.00 | 15.16 | 19.00 | 17.98 | 11.87 | 17.25 | 21.71 | 18.77 |
| SPTB1_HUMAN | 17.91 | 11.69 | 9.88 | 12.22 | 11.18 | 11.01 | 11.61 | 8.43 | 9.55 | 8.52 | 10.12 | 17.25 | 15.80 | 9.42 | 12.16 | 19.35 | 14.38 |
| **PIP_HUMAN** | 16.71 | 15.07 | 13.55 | 15.46 | 12.62 | 16.67 | 12.52 | 10.73 | 11.57 | 13.60 | 22.40 | 11.72 | 17.81 | 13.66 | 17.77 | 16.84 | 19.43 |
| TGM2_HUMAN | 15.26 | 12.99 | 8.17 | 10.34 | 10.71 | 7.77 | 7.70 | 8.90 | 8.17 | 10.44 | 14.73 | 15.02 | 14.06 | 8.22 | 15.48 | 13.48 | 13.08 |
| THOC4_HUMAN | 13.01 | 16.25 | 11.87 | 17.45 | 14.69 | 9.81 | 9.85 | 11.62 | 10.22 | 16.46 | 19.21 | 15.40 | 15.96 | 14.00 | 17.38 | 19.08 | 15.84 |
| PGRP2_HUMAN | 15.20 | 17.87 | 16.46 | 11.22 | 13.98 | 14.57 | 16.13 | 9.76 | 10.89 | 13.85 | 11.76 | 11.48 | 15.49 | 9.70 | 18.32 | 15.67 | 18.46 |

**Supplementary table 1** A table of fully quantitated proteins with the highest standard error (SE) of protein intensity across 17 investigated formalin fixed paraffin embedded breast tissue (BFPT) voxels retrieved by applying the filter_data function of the SpatiaHeatmap R package. A cut-off on SE of the summed protein intensity was set (SE>0.18) to filter the most spatially changed proteins across the BFPT voxels. Bolded are the proteins that were selected for further investigation.

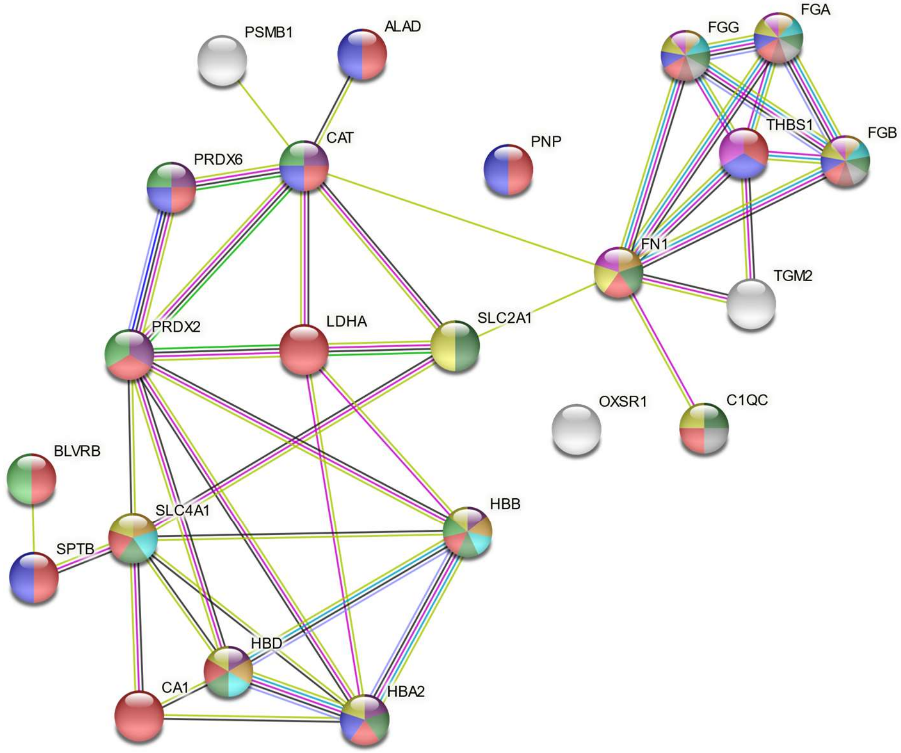


| Colour in STRING map | Type of analysis | term ID | term description | observed gene count | background gene count | strength | false discovery rate | matching proteins in the network (labels) |
|---|---|---|---|---|---|---|---|---|
| DARK VIOLET | GO Process | GO:0042744 | Hydrogen peroxide catabolic process | 6 | 28 | 2.26 | 2.18E-08 | CAT,HBA2,PRDX2,HBB,PRDX6,HBD |
| | GO Process | GO:0045055 | Regulated exocytosis | 11 | 697 | 1.13 | 4.96E-07 | CAT,THBS1,PSMB1,FGB,FGA,HBB,FGG,PRDX6,FN1,PNP,ALAD |
| | GO Process | GO:0010035 | Response to inorganic substance | 10 | 538 | 1.2 | 7.17E-07 | CAT,HBA2,THBS1,PRDX2,FGB,FGA,HBB,FGG,ALAD,LDHA |
| | GO Process | GO:0006950 | Response to stress | 18 | 3485 | 0.64 | 1.09E-06 | CAT,HBA2,THBS1,PSMB1,SLC4A1,PRDX2,FGB,FGA,OXSR1,HBB,FGG,PRDX6,FN1,C1QC,HBD,ALAD,SLC2A1,LDHA |
| | GO Process | GO:0016192 | Vesicle-mediated transport | 14 | 1805 | 0.82 | 2.05E-06 | CAT,HBA2,THBS1,PSMB1,FGB,FGA,HBB,FGG,PRDX6,FN1,PNP,TGM2,ALAD,SPTB |
| | GO Process | GO:0042221 | Response to chemical | 19 | 4333 | 0.57 | 2.05E-06 | CAT,HBA2,THBS1,PSMB1,PRDX2,FGB,FGA,OXSR1,HBB,FGG,PRDX6,FN1,PNP,HBD,ALAD,SLC2A1,CA1,LDHA,SPTB |
| | GO Process | GO:0098869 | Cellular oxidant detoxification | 6 | 90 | 1.75 | 2.05E-06 | CAT,HBA2,PRDX2,HBB,PRDX6,HBD |
| | GO Process | GO:0009636 | Response to toxic substance | 7 | 219 | 1.43 | 4.77E-06 | CAT,HBA2,PRDX2,HBB,PRDX6,HBD,ALAD |
| | GO Process | GO:0006979 | Response to oxidative stress | 8 | 393 | 1.24 | 8.15E-06 | CAT,HBA2,PRDX2,OXSR1,HBB,PRDX6,ALAD,LDHA |
| | GO Process | GO:0006810 | Transport | 18 | 4353 | 0.55 | 1.28E-05 | CAT,HBA2,THBS1,PSMB1,SLC4A1,FGB,FGA,HBB,FGG,PRDX6,FN1,PNP,TGM2,HBD,ALAD,SLC2A1,CA1,SPTB |
| | GO Process | GO:0030193 | Regulation of blood coagulation | 5 | 80 | 1.73 | 2.64E-05 | THBS1,PRDX2,FGB,FGA,FGG |

| | | | | | | | | |
|---|---|---|---|---|---|---|---|---|
| | GO Process | GO:0050896 | Response to stimulus | 22 | 8046 | 0.37 | 2.68E-05 | CAT,HBA2,THBS1,PSMB1,SLC4A1,PRDX2,FGB,FGA,OXSR1,HBB,FGG,PRDX6,FN1,PNP,TGM2,C1QC,HBD,ALAD,SLC2A1,CA1,LDHA,SPTB |
| | GO Process | GO:0070887 | Cellular response to chemical stimulus | 15 | 2919 | 0.64 | 3.15E-05 | CAT,HBA2,THBS1,PSMB1,PRDX2,FGB,OXSR1,HBB,FGG,PRDX6,FN1,HBD,ALAD,SLC2A1,CA1 |
| | GO Process | GO:0050878 | Regulation of body fluid levels | 8 | 509 | 1.13 | 3.81E-05 | THBS1,SLC4A1,PRDX2,FGB,FGA,HBB,FGG,HBD |
| | GO Process | GO:0001775 | Cell activation | 10 | 1075 | 0.9 | 7.32E-05 | CAT,PSMB1,PRDX2,FGB,FGA,HBB,FGG,PRDX6,PNP,ALAD |
| | GO Process | GO:2001237 | Negative regulation of extrinsic apoptotic signaling pathway | 5 | 106 | 1.6 | 7.59E-05 | THBS1,PRDX2,FGB,FGA,FGG |
| | GO Process | GO:0070527 | Platelet aggregation | 4 | 42 | 1.91 | 0.0001 | FGB,FGA,HBB,FGG |
| | GO Process | GO:0015701 | Bicarbonate transport | 4 | 43 | 1.9 | 0.00011 | HBA2,SLC4A1,HBB,CA1 |
| | GO Process | GO:0002576 | Platelet degranulation | 5 | 129 | 1.52 | 0.00018 | THBS1,FGB,FGA,FGG,FN1 |
| | GO Process | GO:2000351 | Regulation of endothelial cell apoptotic process | 4 | 50 | 1.83 | 0.00018 | THBS1,FGB,FGA,FGG |
| | GO Process | GO:0031639 | Plasminogen activation | 3 | 11 | 2.37 | 0.0002 | FGB,FGA,FGG |
| GOLD | GO Process | GO:0042060 | Wound healing | 7 | 439 | 1.13 | 0.0002 | SLC4A1,FGB,FGA,HBB,FGG,FN1,HBD |
| | GO Process | GO:0002376 | Immune system process | 13 | 2481 | 0.65 | 0.00026 | CAT,THBS1,PSMB1,SLC4A1,PRDX2,FGB,FGA,HBB,PRDX6,FN1,PNP,C1QC,ALAD |
| | GO Process | GO:1902041 | Regulation of extrinsic apoptotic signaling pathway via death domain receptors | 4 | 59 | 1.76 | 0.00028 | THBS1,FGB,FGA,FGG |
| | GO Process | GO:0030155 | Regulation of cell adhesion | 8 | 712 | 0.98 | 0.00032 | THBS1,PRDX2,FGB,FGA,FGG,FN1,PNP,TGM2 |
| | GO Process | GO:0010941 | Regulation of cell death | 11 | 1696 | 0.74 | 0.00034 | CAT,HBA2,THBS1,PRDX2,FGB,FGA,HBB,FGG,FN1,TGM2,LDHA |
| CYAN | GO Process | GO:0007596 | Blood coagulation | 6 | 303 | 1.23 | 0.00036 | SLC4A1,FGB,FGA,HBB,FGG,HBD |
| | GO Process | GO:0008152 | Metabolic process | 21 | 8298 | 0.33 | 0.00037 | CAT,HBA2,THBS1,PSMB1,BLVRB,PRDX2,FGB,FGA,OXSR1,HBB,FGG,PRDX6,FN1,PNP,TGM2,HBD,ALAD,SLC2A1,CA1,LDHA,SPTB |
| | GO Process | GO:0034116 | Positive regulation of heterotypic cell-cell adhesion | 3 | 16 | 2.2 | 0.00037 | FGB,FGA,FGG |
| | GO Process | GO:0044237 | Cellular metabolic process | 20 | 7513 | 0.35 | 0.00055 | CAT,HBA2,THBS1,PSMB1,BLVRB,PRDX2,FGA,OXSR1,HBB,FGG,PRDX6,FN1,PNP,TGM2,HBD,ALAD,SLC2A1,CA1,LDHA,SPTB |
| | GO Process | GO:0001666 | Response to hypoxia | 6 | 342 | 1.17 | 0.00057 | CAT,THBS1,PSMB1,ALAD,SLC2A1,LDHA |
| | GO Process | GO:0042730 | Fibrinolysis | 3 | 20 | 2.11 | 0.00059 | FGB,FGA,FGG |
| | GO Process | GO:0000302 | Response to reactive oxygen species | 5 | 198 | 1.33 | 0.00075 | CAT,HBA2,PRDX2,HBB,LDHA |
| | GO Process | GO:0010038 | Response to metal ion | 6 | 367 | 1.14 | 0.00079 | CAT,THBS1,FGB,FGA,FGG,ALAD |
| | GO Process | GO:0051649 | Establishment of localization in cell | 12 | 2375 | 0.63 | 0.00084 | CAT,THBS1,PSMB1,FGB,FGA,HBB,FGG,PRDX6,FN1,PNP,ALAD,SPTB |
| | GO Process | GO:0010810 | Regulation of cell-substrate adhesion | 5 | 212 | 1.3 | 0.00096 | THBS1,FGB,FGA,FGG,FN1 |
| | GO Process | GO:0072378 | Blood coagulation, fibrin clot formation | 3 | 26 | 1.99 | 0.001 | FGB,FGA,FGG |
| | GO Process | GO:0006955 | Immune response | 10 | 1588 | 0.73 | 0.0011 | CAT,THBS1,PSMB1,FGB,FGA,HBB,PRDX6,PNP,C1QC,ALAD |
| | GO Process | GO:0051641 | Cellular localization | 13 | 2967 | 0.57 | 0.0011 | CAT,THBS1,PSMB1,SLC4A1,FGB,FGA,HBB,FGG,PRDX6,FN1,PNP,ALAD,SPTB |
| | GO Process | GO:0002443 | Leukocyte mediated immunity | 7 | 641 | 0.97 | 0.0012 | CAT,PSMB1,HBB,PRDX6,PNP,C1QC,ALAD |
| | GO Process | GO:2000352 | Negative regulation of endothelial cell apoptotic process | 3 | 29 | 1.94 | 0.0013 | FGB,FGA,FGG |
| | GO Process | GO:0045785 | Positive regulation of cell adhesion | 6 | 423 | 1.08 | 0.0014 | FGB,FGA,FGG,FN1,PNP,TGM2 |
| | GO Process | GO:0045907 | Positive regulation of vasoconstriction | 3 | 32 | 1.9 | 0.0016 | FGB,FGA,FGG |
| | GO Process | GO:0002252 | Immune effector process | 8 | 969 | 0.85 | 0.0017 | CAT,PSMB1,PRDX2,HBB,PRDX6,PNP,C1QC,ALAD |

| | | | | | | | | |
|---|---|---|---|---|---|---|---|---|
| | GO Process | GO:0018149 | Peptide cross-linking | 3 | 34 | 1.88 | 0.0019 | THBS1,FN1,TGM2 |
| | GO Process | GO:0042542 | Response to hydrogen peroxide | 4 | 119 | 1.46 | 0.0021 | CAT,HBA2,HBB,LDHA |
| | GO Process | GO:0051716 | Cellular response to stimulus | 18 | 6489 | 0.37 | 0.0021 | CAT,HBA2,THBS1,PSMB1,PRDX2,FGB,FGA,OXSR1,HBB,FGG,PRDX6,FN1,TGM2,HBD,ALAD,SLC2A1,CA1,SPTB |
| | GO Process | GO:0032101 | Regulation of response to external stimulus | 8 | 1013 | 0.83 | 0.0022 | THBS1,PSMB1,PRDX2,FGB,FGA,OXSR1,FGG,TGM2 |
| | GO Process | GO:0071345 | Cellular response to cytokine stimulus | 8 | 1013 | 0.83 | 0.0022 | THBS1,PSMB1,FGB,OXSR1,FGG,FN1,ALAD,CA1 |
| | GO Process | GO:0010811 | Positive regulation of cell-substrate adhesion | 4 | 124 | 1.44 | 0.0023 | FGB,FGA,FGG,FN1 |
| | GO Process | GO:1900026 | Positive regulation of substrate adhesion-dependent cell spreading | 3 | 38 | 1.83 | 0.0024 | FGB,FGA,FGG |
| | GO Process | GO:0007160 | Cell-matrix adhesion | 4 | 127 | 1.43 | 0.0025 | FGB,FGA,FGG,FN1 |
| | GO Process | GO:1902042 | Negative regulation of extrinsic apoptotic signaling pathway via death domain receptors | 3 | 39 | 1.82 | 0.0025 | FGB,FGA,FGG |
| | GO Process | GO:0043312 | Neutrophil degranulation | 6 | 484 | 1.02 | 0.0026 | CAT,PSMB1,HBB,PRDX6,PNP,ALAD |
| | GO Process | GO:0062197 | Cellular response to chemical stress | 5 | 293 | 1.16 | 0.0031 | CAT,PRDX2,OXSR1,PRDX6,SLC2A1 |
| | GO Process | GO:0051592 | Response to calcium ion | 4 | 150 | 1.36 | 0.0038 | THBS1,FGB,FGA,FGG |
| | GO Process | GO:0065008 | Regulation of biological quality | 14 | 4042 | 0.47 | 0.0041 | THBS1,PSMB1,SLC4A1,PRDX2,FGB,FGA,HBB,FGG,PRDX6,FN1,TGM2,HBD,SLC2A1,SPTB |
| | GO Process | GO:0010770 | Positive regulation of cell morphogenesis involved in differentiation | 4 | 159 | 1.33 | 0.0046 | FGB,FGA,FGG,FN1 |
| | GO Process | GO:0042981 | Regulation of apoptotic process | 9 | 1550 | 0.69 | 0.0047 | CAT,THBS1,PRDX2,FGB,FGA,FGG,FN1,TGM2,LDHA |
| | GO Process | GO:0043152 | Induction of bacterial agglutination | 2 | 6 | 2.45 | 0.0047 | FGB,FGA |
| | GO Process | GO:0030198 | Extracellular matrix organization | 5 | 338 | 1.1 | 0.0051 | THBS1,FGB,FGA,FGG,FN1 |
| | GO Process | GO:0010033 | Response to organic substance | 12 | 3011 | 0.53 | 0.0054 | CAT,THBS1,PSMB1,PRDX2,FGB,OXSR1,FGG,FN1,ALAD,SLC2A1,CA1,LDHA |
| | GO Process | GO:0009056 | Catabolic process | 10 | 2042 | 0.62 | 0.0058 | CAT,HBA2,PSMB1,BLVRB,PRDX2,HBB,PRDX6,PNP,HBD,LDHA |
| | GO Process | GO:0043066 | Negative regulation of apoptotic process | 7 | 893 | 0.82 | 0.0063 | CAT,THBS1,PRDX2,FGB,FGA,FGG,FN1 |
| | GO Process | GO:0032102 | Negative regulation of response to external stimulus | 5 | 367 | 1.06 | 0.0069 | THBS1,PRDX2,FGB,FGA,FGG |
| | GO Process | GO:0045321 | Leukocyte activation | 7 | 929 | 0.81 | 0.0078 | CAT,PSMB1,PRDX2,HBB,PRDX6,PNP,ALAD |
| | GO Process | GO:0044248 | Cellular catabolic process | 9 | 1758 | 0.64 | 0.0105 | CAT,HBA2,PSMB1,BLVRB,PRDX2,HBB,PRDX6,PNP,HBD |
| | GO Process | GO:0033197 | Response to vitamin e | 2 | 11 | 2.19 | 0.0107 | CAT,ALAD |
| | GO Process | GO:0090276 | Regulation of peptide hormone secretion | 4 | 215 | 1.2 | 0.0118 | FGB,FGA,FGG,SLC2A1 |
| | GO Process | GO:0048584 | Positive regulation of response to stimulus | 10 | 2257 | 0.58 | 0.0119 | CAT,THBS1,PSMB1,PRDX2,FGB,FGA,OXSR1,FGG,TGM2,C1QC |
| | GO Process | GO:0022407 | Regulation of cell-cell adhesion | 5 | 424 | 1 | 0.0122 | PRDX2,FGB,FGA,FGG,PNP |
| | GO Process | GO:0009605 | Response to external stimulus | 10 | 2310 | 0.57 | 0.0143 | CAT,THBS1,PRDX2,FGB,FGA,C1QC,ALAD,SLC2A1,LDHA,SPTB |
| | GO Process | GO:1902533 | Positive regulation of intracellular signal transduction | 7 | 1041 | 0.76 | 0.0143 | CAT,THBS1,PRDX2,FGB,FGA,FGG,TGM2 |
| | GO Process | GO:0043408 | Regulation of mapk cascade | 6 | 725 | 0.85 | 0.0153 | THBS1,PRDX2,FGB,FGA,FGG,FN1 |
| | GO Process | GO:0051258 | Protein polymerization | 3 | 87 | 1.47 | 0.0153 | FGB,FGA,FGG |
| | GO Process | GO:0045595 | Regulation of cell differentiation | 9 | 1874 | 0.61 | 0.0157 | THBS1,PSMB1,PRDX2,FGB,FGA,FGG,FN1,PNP,C1QC |

| | | | | | | | | |
|---|---|---|---|---|---|---|---|---|
| | GO Process | GO:0048518 | Positive regulation of biological process | 16 | 6112 | 0.35 | 0.0174 | CAT,HBA2,THBS1,PSMB1,PRDX2,FGB,FGA,OXSR1,HBB,FGG,PRDX6,FN1,PNP,TGM2,C1QC,LDHA |
| | GO Process | GO:0015711 | Organic anion transport | 5 | 465 | 0.96 | 0.0175 | HBA2,SLC4A1,HBB,SLC2A1,CA1 |
| | GO Process | GO:0045921 | Positive regulation of exocytosis | 3 | 92 | 1.44 | 0.0175 | FGB,FGA,FGG |
| | GO Process | GO:0002224 | Toll-like receptor signaling pathway | 3 | 100 | 1.41 | 0.0219 | FGB,FGA,FGG |
| | GO Process | GO:0090277 | Positive regulation of peptide hormone secretion | 3 | 100 | 1.41 | 0.0219 | FGB,FGA,FGG |
| | GO Process | GO:1903706 | Regulation of hemopoiesis | 5 | 493 | 0.94 | 0.0223 | THBS1,PSMB1,PRDX2,PNP,C1QC |
| | GO Process | GO:0009628 | Response to abiotic stimulus | 7 | 1147 | 0.72 | 0.0238 | CAT,THBS1,PSMB1,OXSR1,ALAD,SLC2A1,LDHA |
| | GO Process | GO:0022409 | Positive regulation of cell-cell adhesion | 4 | 272 | 1.1 | 0.0249 | FGB,FGA,FGG,PNP |
| | GO Process | GO:0050794 | Regulation of cellular process | 21 | 10932 | 0.21 | 0.0249 | CAT,HBA2,THBS1,PSMB1,SLC4A1,PRDX2,FGB,FGA,OXSR1,HBB,FGG,PRDX6,FN1,PNP,TGM2,C1QC,ALAD,SLC2A1,CA1,LDHA,SPTB |
| | GO Process | GO:0065007 | Biological regulation | 22 | 12171 | 0.19 | 0.0249 | CAT,HBA2,THBS1,PSMB1,SLC4A1,PRDX2,FGB,FGA,OXSR1,HBB,FGG,PRDX6,FN1,PNP,TGM2,C1QC,HBD,ALAD,SLC2A1,CA1,LDHA,SPTB |
| | GO Process | GO:0048522 | Positive regulation of cellular process | 15 | 5579 | 0.36 | 0.025 | CAT,HBA2,THBS1,PSMB1,PRDX2,FGB,FGA,OXSR1,HBB,FGG,PRDX6,FN1,PNP,TGM2,LDHA |
| | GO Process | GO:1901700 | Response to oxygen-containing compound | 8 | 1567 | 0.64 | 0.025 | CAT,HBA2,THBS1,PRDX2,HBB,ALAD,SLC2A1,LDHA |
| | GO Process | GO:0090066 | Regulation of anatomical structure size | 5 | 516 | 0.92 | 0.026 | FGB,FGA,FGG,FN1,SPTB |
| | GO Process | GO:0009725 | Response to hormone | 6 | 849 | 0.78 | 0.0315 | CAT,THBS1,FGB,ALAD,SLC2A1,LDHA |
| | GO Process | GO:0042325 | Regulation of phosphorylation | 8 | 1626 | 0.62 | 0.0315 | THBS1,SLC4A1,PRDX2,FGB,FGA,OXSR1,FGG,FN1 |
| | GO Process | GO:0070372 | Regulation of erk1 and erk2 cascade | 4 | 292 | 1.07 | 0.0315 | FGB,FGA,FGG,FN1 |
| | GO Process | GO:0043410 | Positive regulation of mapk cascade | 5 | 543 | 0.89 | 0.0319 | THBS1,PRDX2,FGB,FGA,FGG |
| | GO Process | GO:0010288 | Response to lead ion | 2 | 23 | 1.87 | 0.0331 | CAT,ALAD |
| | GO Process | GO:0051241 | Negative regulation of multicellular organismal process | 7 | 1231 | 0.68 | 0.0334 | THBS1,PRDX2,FGB,FGA,FGG,FN1,C1QC |
| | GO Process | GO:0009967 | Positive regulation of signal transduction | 8 | 1654 | 0.61 | 0.0341 | CAT,THBS1,PSMB1,PRDX2,FGB,FGA,FGG,TGM2 |
| | GO Process | GO:0030194 | Positive regulation of blood coagulation | 2 | 28 | 1.78 | 0.0457 | THBS1,PRDX2 |
| | GO Process | GO:0007155 | Cell adhesion | 6 | 925 | 0.74 | 0.0458 | THBS1,FGB,FGA,HBB,FGG,FN1 |
| | GO Process | GO:0050708 | Regulation of protein secretion | 4 | 333 | 1.01 | 0.0469 | FGB,FGA,FGG,SLC2A1 |
| | GO Process | GO:1901564 | Organonitrogen compound metabolic process | 14 | 5244 | 0.36 | 0.0478 | CAT,THBS1,PSMB1,BLVRB,FGB,FGA,OXSR1,FGG,FN1,PNP,TGM2,ALAD,LDHA,SPTB |
| | GO Function | GO:0005201 | Extracellular matrix structural constituent | 5 | 119 | 1.55 | 0.00093 | THBS1,FGB,FGA,FGG,FN1 |
| | GO Function | GO:0005344 | Oxygen carrier activity | 3 | 14 | 2.26 | 0.00093 | HBA2,HBB,HBD |
| | GO Function | GO:0030492 | Hemoglobin binding | 3 | 9 | 2.45 | 0.00093 | SLC4A1,HBB,HBD |
| | GO Function | GO:0042802 | Identical protein binding | 12 | 1896 | 0.73 | 0.00093 | CAT,THBS1,SLC4A1,OXSR1,FGG,PRDX6,FN1,PNP,TGM2,ALAD,SLC2A1,LDHA |
| | GO Function | GO:0019825 | Oxygen binding | 3 | 36 | 1.85 | 0.0081 | HBA2,HBB,HBD |
| | GO Function | GO:0004601 | Peroxidase activity | 3 | 41 | 1.79 | 0.0098 | CAT,PRDX2,PRDX6 |
| | GO Function | GO:0020037 | Heme binding | 4 | 134 | 1.4 | 0.0098 | CAT,HBA2,HBB,HBD |
| | GO Function | GO:0031721 | Hemoglobin alpha binding | 2 | 5 | 2.53 | 0.0098 | HBB,HBD |
| | GO Function | GO:0051920 | Peroxiredoxin activity | 2 | 7 | 2.39 | 0.0139 | PRDX2,PRDX6 |
| | GO Function | GO:0005198 | Structural molecule activity | 6 | 635 | 0.91 | 0.0203 | THBS1,FGB,FGA,FGG,FN1,SPTB |

| | | | | | | | | |
|---|---|---|---|---|---|---|---|---|
| | GO Component | GO:0005615 | Extracellular space | 22 | 3195 | 0.77 | 9.67E-14 | CAT,HBA2,THBS1,PSMB1,SLC4A1,BLVRB,PRDX2,FGB,FGA,OXSR1,HBB,FGG,PRDX6,FN1,PNP,TGM2,C1QC,HBD,ALAD,SLC2A1,CA1,LDHA |
| | GO Component | GO:0070062 | Extracellular exosome | 20 | 2099 | 0.91 | 9.67E-14 | CAT,HBA2,THBS1,PSMB1,SLC4A1,BLVRB,PRDX2,FGB,FGA,OXSR1,HBB,FGG,PRDX6,FN1,PNP,TGM2,ALAD,SLC2A1,CA1,LDHA |
| DARK GREEN | GO Component | GO:0072562 | Blood microparticle | 10 | 115 | 1.87 | 9.67E-14 | HBA2,SLC4A1,FGB,FGA,HBB,FGG,FN1,C1QC,HBD,SLC2A1 |
| | GO Component | GO:0005577 | Fibrinogen complex | 5 | 8 | 2.73 | 4.14E-10 | THBS1,FGB,FGA,FGG,FN1 |
| | GO Component | GO:0034774 | Secretory granule lumen | 10 | 324 | 1.42 | 4.14E-10 | CAT,THBS1,PSMB1,FGB,FGA,FGG,PRDX6,FN1,PNP,ALAD |
| | GO Component | GO:0030141 | Secretory granule | 11 | 845 | 1.04 | 1.23E-07 | CAT,THBS1,PSMB1,FGB,FGA,HBB,FGG,PRDX6,FN1,PNP,ALAD |
| | GO Component | GO:0031093 | Platelet alpha granule lumen | 5 | 68 | 1.8 | 2.42E-06 | THBS1,FGB,FGA,FGG,FN1 |
| | GO Component | GO:0062023 | Collagen-containing extracellular matrix | 7 | 396 | 1.18 | 2.90E-05 | THBS1,FGB,FGA,FGG,FN1,TGM2,C1QC |
| | GO Component | GO:1904813 | ficolin-1-rich granule lumen | 5 | 125 | 1.53 | 3.64E-05 | CAT,PSMB1,HBB,PNP,ALAD |
| | GO Component | GO:0031410 | Cytoplasmic vesicle | 13 | 2386 | 0.67 | 4.18E-05 | CAT,HBA2,THBS1,PSMB1,FGB,FGA,HBB,FGG,PRDX6,FN1,PNP,ALAD,SLC2A1 |
| | GO Component | GO:0031838 | Haptoglobin-hemoglobin complex | 3 | 11 | 2.37 | 4.18E-05 | HBA2,HBB,HBD |
| | GO Component | GO:0005833 | Hemoglobin complex | 3 | 12 | 2.33 | 4.92E-05 | HBA2,HBB,HBD |
| | GO Component | GO:0005829 | Cytosol | 16 | 5193 | 0.42 | 0.0014 | CAT,HBA2,PSMB1,BLVRB,PRDX2,OXSR1,HBB,PRDX6,PNP,TGM2,HBD,ALAD,SLC2A1,CA1,LDHA,SPTB |
| | GO Component | GO:0005938 | Cell cortex | 5 | 292 | 1.16 | 0.0014 | SLC4A1,FGB,FGA,SLC2A1,SPTB |
| | GO Component | GO:0098552 | Side of membrane | 6 | 531 | 0.98 | 0.0018 | THBS1,SLC4A1,FGB,FGA,FGG,SPTB |
| | GO Component | GO:0005737 | Cytoplasm | 22 | 11428 | 0.21 | 0.0047 | CAT,HBA2,THBS1,PSMB1,SLC4A1,BLVRB,PRDX2,FGB,FGA,OXSR1,HBB,FGG,PRDX6,FN1,PNP,TGM2,HBD,ALAD,SLC2A1,CA1,LDHA,SPTB |
| | GO Component | GO:0030863 | Cortical cytoskeleton | 3 | 96 | 1.42 | 0.0125 | SLC4A1,SLC2A1,SPTB |
| | GO Component | GO:0071682 | Endocytic vesicle lumen | 2 | 20 | 1.93 | 0.0176 | HBA2,HBB |
| | GO Component | GO:0005788 | Endoplasmic reticulum lumen | 4 | 308 | 1.04 | 0.0249 | THBS1,FGA,FGG,FN1 |
| | GO Component | GO:0009897 | External side of plasma membrane | 4 | 331 | 1.01 | 0.0315 | THBS1,FGB,FGA,FGG |
| | GO Component | GO:0012505 | Endomembrane system | 13 | 4542 | 0.39 | 0.0315 | CAT,THBS1,PSMB1,FGB,FGA,HBB,FGG,PRDX6,FN1,PNP,TGM2,ALAD,SLC2A1 |
| | STRING clusters | CL:21831 | Hemoglobin complex, and glycophorin a | 4 | 19 | 2.25 | 5.72E-05 | HBA2,SLC4A1,HBB,HBD |
| | STRING clusters | CL:19735 | COVID-19, thrombosis and anticoagulation | 3 | 6 | 2.63 | 0.00018 | FGB,FGA,FGG |
| | STRING clusters | CL:21835 | Hemoglobin complex | 3 | 5 | 2.71 | 0.00018 | HBA2,HBB,HBD |
| | STRING clusters | CL:13062 | Glutathione metabolism, and Antioxidant | 3 | 56 | 1.66 | 0.0154 | CAT,PRDX2,PRDX6 |
| | STRING clusters | CL:19706 | Mixed, incl. complement and coagulation cascades, and lipoprotein particle | 4 | 182 | 1.27 | 0.0196 | FGB,FGA,FGG,C1QC |
| | STRING clusters | CL:13136 | Mixed, incl. thioredoxin peroxidase activity, and superoxide dismutase activity | 2 | 11 | 2.19 | 0.0273 | CAT,PRDX2 |

| | | | | | | | | |
|---|---|---|---|---|---|---|---|---|
| GREY | KEGG | hsa04610 | Complement and coagulation cascades | 4 | 82 | 1.62 | 0.00097 | FGB,FGA,FGG,C1QC |
| | KEGG | hsa05144 | Malaria | 3 | 46 | 1.74 | 0.0042 | HBA2,THBS1,HBB |
| BROWN | KEGG | hsa04611 | Platelet activation | 3 | 122 | 1.32 | 0.0458 | FGB,FGA,FGG |
| | Reactome | HSA-354194 | GRB2:SOS provides linkage to MAPK signaling for Integrins | 4 | 15 | 2.36 | 1.20E-05 | FGB,FGA,FGG,FN1 |
| | Reactome | HSA-372708 | p130Cas linkage to MAPK signaling for integrins | 4 | 15 | 2.36 | 1.20E-05 | FGB,FGA,FGG,FN1 |
| | Reactome | HSA-216083 | Integrin cell surface interactions | 5 | 85 | 1.7 | 3.22E-05 | THBS1,FGB,FGA,FGG,FN1 |
| | Reactome | HSA-168249 | Innate Immune System | 10 | 1025 | 0.92 | 4.30E-05 | CAT,PSMB1,FGB,FGA,HBB,FGG,PRDX6,PNP,C1QC,ALAD |
| | Reactome | HSA-6802948 | Signaling by high-kinase activity BRAF mutants | 4 | 36 | 1.98 | 4.64E-05 | FGB,FGA,FGG,FN1 |
| | Reactome | HSA-1247673 | Erythrocytes take up oxygen and release carbon dioxide | 3 | 8 | 2.5 | 5.36E-05 | SLC4A1,HBB,CA1 |
| | Reactome | HSA-5674135 | MAP2K and MAPK activation | 4 | 40 | 1.93 | 5.36E-05 | FGB,FGA,FGG,FN1 |
| | Reactome | HSA-9656223 | Signaling by RAF1 mutants | 4 | 41 | 1.92 | 5.36E-05 | FGB,FGA,FGG,FN1 |
| | Reactome | HSA-114608 | Platelet degranulation | 5 | 127 | 1.52 | 5.83E-05 | THBS1,FGB,FGA,FGG,FN1 |
| | Reactome | HSA-6802946 | Signaling by moderate kinase activity BRAF mutants | 4 | 45 | 1.88 | 5.83E-05 | FGB,FGA,FGG,FN1 |
| | Reactome | HSA-6802955 | Paradoxical activation of RAF signaling by kinase inactive BRAF | 4 | 45 | 1.88 | 5.83E-05 | FGB,FGA,FGG,FN1 |
| | Reactome | HSA-9649948 | Signaling downstream of RAS mutants | 4 | 45 | 1.88 | 5.83E-05 | FGB,FGA,FGG,FN1 |
| | Reactome | HSA-168256 | Immune System | 12 | 1956 | 0.72 | 6.42E-05 | CAT,PSMB1,FGB,FGA,HBB,FGG,PRDX6,FN1,PNP,C1QC,ALAD,CA1 |
| | Reactome | HSA-1237044 | Erythrocytes take up carbon dioxide and release oxygen | 3 | 12 | 2.33 | 7.69E-05 | SLC4A1,HBB,CA1 |
| | Reactome | HSA-5673001 | RAF/MAP kinase cascade | 6 | 274 | 1.27 | 7.69E-05 | PSMB1,FGB,FGA,FGG,FN1,SPTB |
| | Reactome | HSA-6802952 | Signaling by BRAF and RAF fusions | 4 | 65 | 1.72 | 0.00012 | FGB,FGA,FGG,FN1 |
| | Reactome | HSA-5686938 | Regulation of TLR by endogenous ligand | 3 | 19 | 2.13 | 0.00019 | FGB,FGA,FGG |
| | Reactome | HSA-140875 | Common Pathway of Fibrin Clot Formation | 3 | 22 | 2.06 | 0.00027 | FGB,FGA,FGG |
| | Reactome | HSA-109582 | Hemostasis | 7 | 605 | 0.99 | 0.00036 | THBS1,FGB,FGA,HBB,FGG,FN1,HBD |
| | Reactome | HSA-3299685 | Detoxification of Reactive Oxygen Species | 3 | 37 | 1.84 | 0.001 | CAT,PRDX2,PRDX6 |
| | Reactome | HSA-6798695 | Neutrophil degranulation | 6 | 473 | 1.03 | 0.0011 | CAT,PSMB1,HBB,PRDX6,PNP,ALAD |
| | Reactome | HSA-1643685 | Disease | 9 | 1548 | 0.69 | 0.0024 | THBS1,PSMB1,SLC4A1,PRDX2,FGB,FGA,FGG,FN1,SLC2A1 |
| | Reactome | HSA-5663202 | Diseases of signal transduction by growth factor receptors and second messengers | 5 | 392 | 1.04 | 0.0053 | PSMB1,FGB,FGA,FGG,FN1 |
| | Reactome | HSA-8957275 | Post-translational protein phosphorylation | 3 | 107 | 1.38 | 0.0174 | FGA,FGG,FN1 |
| | Reactome | HSA-381426 | Regulation of Insulin-like Growth Factor (IGF) transport and uptake by Insulin-like Growth Factor Binding Proteins (IGFBPs) | 3 | 124 | 1.31 | 0.0259 | FGA,FGG,FN1 |
| | Reactome | HSA-189445 | Metabolism of porphyrins | 2 | 27 | 1.8 | 0.0308 | BLVRB,ALAD |
| | Reactome | HSA-3000170 | Syndecan interactions | 2 | 27 | 1.8 | 0.0308 | THBS1,FN1 |
| | WikiPathways | WP15 | Selenium micronutrient network | 7 | 86 | 1.84 | 6.75E-09 | CAT,HBA2,PRDX2,FGB,FGA,HBB,FGG |

| | | | | | | | | |
|---|---|---|---|---|---|---|---|---|
| | WikiPathways | WP176 | Folate metabolism | 6 | 69 | 1.87 | 8.35E-08 | CAT,HBA2,FGB,FGA,HBB,FGG |
| | WikiPathways | WP3888 | VEGFA-VEGFR2 signaling pathway | 9 | 428 | 1.25 | 1.77E-07 | PRDX2,FGB,FGA,FGG,PRDX6,FN1,PNP,HBD,LDHA |
| | WikiPathways | WP4927 | COVID-19, thrombosis and anticoagulation | 3 | 7 | 2.56 | 2.86E-05 | FGB,FGA,FGG |
| | WikiPathways | WP272 | Blood clotting cascade | 3 | 23 | 2.05 | 0.00049 | FGB,FGA,FGG |
| | WikiPathways | WP2806 | Complement system | 4 | 97 | 1.55 | 0.00062 | THBS1,FGB,FGA,FGG |
| | WikiPathways | WP4136 | Fibrin complement receptor 3 signaling pathway | 3 | 41 | 1.79 | 0.0018 | FGB,FGA,FGG |
| | WikiPathways | WP2456 | HIF1A and PPARG regulation of glycolysis | 2 | 8 | 2.33 | 0.005 | SLC2A1,LDHA |
| | WikiPathways | WP4629 | Aerobic glycolysis | 2 | 12 | 2.15 | 0.009 | SLC2A1,LDHA |
| | WikiPathways | WP1946 | Cori cycle | 2 | 17 | 2 | 0.0151 | SLC2A1,LDHA |
| | WikiPathways | WP4206 | Hereditary leiomyomatosis and renal cell carcinoma pathway | 2 | 20 | 1.93 | 0.0185 | SLC2A1,LDHA |
| | WikiPathways | WP2911 | miRNA targets in ECM and membrane receptors | 2 | 22 | 1.89 | 0.0202 | THBS1,FN1 |
| | WikiPathways | WP2884 | NRF2 pathway | 3 | 143 | 1.25 | 0.0334 | BLVRB,PRDX6,SLC2A1 |
| | WikiPathways | WP453 | Inflammatory response pathway | 2 | 30 | 1.75 | 0.0334 | THBS1,FN1 |
| | WikiPathways | WP3614 | Photodynamic therapy-induced HIF-1 survival signaling | 2 | 37 | 1.66 | 0.043 | SLC2A1,LDHA |
| | Monarch HPO | HP:0011900 | Hypofibrinogenemia | 5 | 22 | 2.29 | 1.10E-06 | SLC4A1,FGB,FGA,FGG,SPTB |
| | Monarch HPO | HP:0001871 | Abnormality of blood and blood-forming tissues | 12 | 1216 | 0.92 | 1.13E-05 | CAT,SLC4A1,FGB,FGA,HBB,FGG,FN1,PNP,HBD,ALAD,SLC2A1,SPTB |
| | Monarch HPO | HP:0001878 | Hemolytic anemia | 6 | 102 | 1.7 | 1.13E-05 | SLC4A1,HBB,PNP,ALAD,SLC2A1,SPTB |
| | Monarch HPO | HP:0004936 | Venous thrombosis | 5 | 65 | 1.82 | 3.20E-05 | SLC4A1,FGB,FGA,HBB,FGG |
| | Monarch HPO | HP:0001928 | Abnormality of coagulation | 6 | 171 | 1.47 | 7.34E-05 | SLC4A1,FGB,FGA,HBB,FGG,SPTB |
| | Monarch HPO | HP:0012223 | Splenic rupture | 3 | 4 | 2.8 | 7.34E-05 | FGB,FGA,FGG |
| | Monarch HPO | HP:0002715 | Abnormality of the immune system | 12 | 1700 | 0.78 | 0.00012 | CAT,SLC4A1,FGB,FGA,HBB,FGG,PNP,C1QC,ALAD,SLC2A1,LDHA,SPTB |
| | Monarch HPO | HP:0001877 | Abnormal erythrocyte morphology | 8 | 556 | 1.09 | 0.00015 | CAT,SLC4A1,HBB,PNP,HBD,ALAD,SLC2A1,SPTB |
| | Monarch HPO | HP:0001923 | Reticulocytosis | 4 | 38 | 1.95 | 0.00015 | SLC4A1,HBB,SLC2A1,SPTB |
| | Monarch HPO | HP:0005525 | Spontaneous hemolytic crises | 3 | 7 | 2.56 | 0.00015 | SLC4A1,SLC2A1,SPTB |
| | Monarch HPO | HP:0100763 | Abnormality of the lymphatic system | 8 | 577 | 1.07 | 0.00015 | SLC4A1,FGB,FGA,HBB,FGG,PNP,SLC2A1,SPTB |
| | Monarch HPO | HP:0000225 | Gingival bleeding | 4 | 45 | 1.88 | 0.00021 | CAT,FGB,FGA,FGG |
| | Monarch HPO | HP:0001903 | Anemia | 7 | 426 | 1.15 | 0.00025 | SLC4A1,HBB,PNP,HBD,ALAD,SLC2A1,SPTB |
| | Monarch HPO | HP:0004447 | Poikilocytosis | 4 | 48 | 1.85 | 0.00025 | SLC4A1,HBB,SLC2A1,SPTB |
| | Monarch HPO | HP:0025066 | Decreased mean corpuscular volume | 3 | 10 | 2.41 | 0.00025 | SLC4A1,HBB,SPTB |
| | Monarch HPO | HP:0400008 | Menometrorrhagia | 3 | 10 | 2.41 | 0.00025 | FGB,FGA,FGG |
| | Monarch HPO | HP:0004446 | Stomatocytosis | 3 | 13 | 2.29 | 0.00041 | SLC4A1,SLC2A1,SPTB |
| | Monarch HPO | HP:0001342 | Cerebral hemorrhage | 4 | 61 | 1.75 | 0.00046 | FGB,FGA,FGG,FN1 |
| | Monarch HPO | HP:0001939 | Abnormality of metabolism/homeostasis | 12 | 2167 | 0.67 | 0.00063 | CAT,SLC4A1,FGB,FGA,HBB,FGG,FN1,PNP,ALAD,SLC2A1,LDHA,SPTB |
| | Monarch HPO | HP:0025546 | Abnormal mean corpuscular hemoglobin concentration | 3 | 16 | 2.2 | 0.00063 | SLC4A1,HBB,SPTB |
| | Monarch HPO | HP:0100724 | Hypercoagulability | 3 | 19 | 2.13 | 0.00089 | SLC4A1,HBB,SPTB |

| | | | | | | | | |
|---|---|---|---|---|---|---|---|---|
| | Monarch HPO | EFO:0004747 | Protein measurement | 18 | 5855 | 0.42 | 0.00095 | CAT,PSMB1,SLC4A1,FGB,FGA,OXSR1,HBB,FGG,PRDX6,FN1,PNP,TGM2,C1QC,HBD,SLC2A1,CA1,LDHA,SPTB |
| | Monarch HPO | HP:0001978 | Extramedullary hematopoiesis | 3 | 20 | 2.11 | 0.00095 | SLC4A1,HBB,SPTB |
| | Monarch HPO | EFO:0004272 | Anemia (phenotype) | 5 | 189 | 1.35 | 0.00097 | CAT,SLC4A1,HBB,HBD,SPTB |
| | Monarch HPO | HP:0005268 | Miscarriage | 3 | 21 | 2.08 | 0.001 | FGB,FGA,FGG |
| | Monarch HPO | HP:0012379 | Abnormal enzyme/coenzyme activity | 5 | 198 | 1.33 | 0.0011 | CAT,SLC4A1,HBB,ALAD,LDHA |
| | Monarch HPO | EFO:0004503 | Hematological measurement | 16 | 4594 | 0.47 | 0.0012 | SLC4A1,BLVRB,PRDX2,FGB,FGA,OXSR1,HBB,FGG,FN1,TGM2,HBD,ALAD,SLC2A1,CA1,LDHA,SPTB |
| | Monarch HPO | HP:0002904 | Hyperbilirubinemia | 4 | 89 | 1.58 | 0.0012 | SLC4A1,HBB,SLC2A1,SPTB |
| | Monarch HPO | HP:0001744 | Splenomegaly | 6 | 383 | 1.12 | 0.0013 | SLC4A1,FGA,HBB,PNP,SLC2A1,SPTB |
| | Monarch HPO | HP:0100659 | Abnormal cerebral vascular morphology | 6 | 381 | 1.13 | 0.0013 | FGB,FGA,HBB,FGG,FN1,PNP |
| | Monarch HPO | HP:0001935 | Microcytic anemia | 3 | 28 | 1.96 | 0.0017 | CAT,HBB,HBD |
| | Monarch HPO | HP:0011277 | Abnormality of the urinary system physiology | 8 | 931 | 0.86 | 0.0019 | SLC4A1,FGA,HBB,FN1,PNP,C1QC,ALAD,LDHA |
| | Monarch HPO | HP:0002597 | Abnormality of the vasculature | 10 | 1673 | 0.71 | 0.0022 | CAT,SLC4A1,FGB,FGA,HBB,FGG,FN1,PNP,SLC2A1,SPTB |
| | Monarch HPO | HP:0001386 | Joint swelling | 3 | 35 | 1.86 | 0.0027 | FGB,FGA,FGG |
| | Monarch HPO | HP:0004364 | Abnormal circulating nitrogen compound concentration | 6 | 447 | 1.06 | 0.0027 | SLC4A1,HBB,PNP,SLC2A1,LDHA,SPTB |
| | Monarch HPO | HP:0012649 | Increased inflammatory response | 8 | 984 | 0.84 | 0.0027 | CAT,SLC4A1,FGA,HBB,PNP,C1QC,LDHA,SPTB |
| | Monarch HPO | HP:0003111 | Abnormal blood ion concentration | 5 | 277 | 1.19 | 0.0037 | SLC4A1,HBB,ALAD,SLC2A1,LDHA |
| | Monarch HPO | HP:0000980 | Pallor | 4 | 135 | 1.4 | 0.0042 | SLC4A1,HBB,SLC2A1,SPTB |
| | Monarch HPO | HP:0002012 | Abnormality of the abdominal organs | 8 | 1069 | 0.8 | 0.0042 | SLC4A1,FGB,FGA,HBB,FGG,PNP,SLC2A1,SPTB |
| | Monarch HPO | HP:0004444 | Spherocytosis | 2 | 5 | 2.53 | 0.0057 | SLC4A1,SPTB |
| | Monarch HPO | HP:0001743 | Abnormality of the spleen | 4 | 152 | 1.35 | 0.006 | FGB,FGA,HBB,FGG |
| | Monarch HPO | EFO:0004528 | Mean corpuscular hemoglobin concentration | 6 | 545 | 0.97 | 0.0063 | SLC4A1,OXSR1,HBB,SLC2A1,CA1,SPTB |
| | Monarch HPO | HP:0001081 | Cholelithiasis | 3 | 52 | 1.69 | 0.0068 | SLC4A1,HBB,SPTB |
| | Monarch HPO | EFO:0004623 | Fibrinogen measurement | 3 | 54 | 1.67 | 0.0069 | FGB,FGA,FGG |
| | Monarch HPO | HP:0004870 | Chronic hemolytic anemia | 2 | 6 | 2.45 | 0.0069 | HBB,SPTB |
| | Monarch HPO | HP:0008341 | Distal renal tubular acidosis | 2 | 6 | 2.45 | 0.0069 | SLC4A1,FN1 |
| | Monarch HPO | HP:0010972 | Anemia of inadequate production | 4 | 160 | 1.33 | 0.0069 | CAT,SLC4A1,HBB,HBD |
| | Monarch HPO | HP:0012211 | Abnormal renal physiology | 6 | 558 | 0.96 | 0.0069 | SLC4A1,FGA,HBB,FN1,C1QC,LDHA |
| | Monarch HPO | HP:0004297 | Abnormality of the biliary system | 4 | 164 | 1.32 | 0.007 | SLC4A1,FGA,HBB,SPTB |
| | Monarch HPO | HP:0025142 | Constitutional symptom | 7 | 864 | 0.84 | 0.0074 | CAT,SLC4A1,HBB,ALAD,SLC2A1,LDHA,SPTB |
| | Monarch HPO | HP:0002027 | Abdominal pain | 4 | 172 | 1.3 | 0.008 | SLC4A1,HBB,ALAD,SPTB |
| | Monarch HPO | HP:0045040 | Abnormal lactate dehydrogenase level | 3 | 58 | 1.64 | 0.008 | SLC4A1,HBB,LDHA |
| | Monarch HPO | EFO:0010700 | Reticulocyte measurement | 7 | 887 | 0.83 | 0.0083 | SLC4A1,BLVRB,OXSR1,ALAD,SLC2A1,CA1,SPTB |
| | Monarch HPO | HP:0000952 | Jaundice | 4 | 175 | 1.29 | 0.0083 | SLC4A1,HBB,SLC2A1,SPTB |
| | Monarch HPO | HP:0000079 | Abnormality of the urinary system | 9 | 1657 | 0.66 | 0.0091 | SLC4A1,FGA,HBB,FN1,PNP,C1QC,ALAD,SLC2A1,LDHA |
| | Monarch HPO | HP:0004804 | Congenital hemolytic anemia | 2 | 8 | 2.33 | 0.0091 | SLC4A1,SPTB |
| | Monarch HPO | HP:0001046 | Intermittent jaundice | 2 | 9 | 2.28 | 0.0101 | SLC4A1,SPTB |

| | | | | | | | | |
|---|---|---|---|---|---|---|---|---|
| | Monarch HPO | HP:0001930 | Nonspherocytic hemolytic anemia | 2 | 9 | 2.28 | 0.0101 | SLC4A1,HBB |
| | Monarch HPO | HP:0004445 | Elliptocytosis | 2 | 9 | 2.28 | 0.0101 | SLC4A1,SPTB |
| | Monarch HPO | HP:0003110 | Abnormality of urine homeostasis | 6 | 638 | 0.9 | 0.0103 | FGA,HBB,FN1,PNP,ALAD,LDHA |
| | Monarch HPO | EFO:0010701 | Mean reticulocyte volume | 5 | 408 | 1.02 | 0.013 | SLC4A1,OXSR1,SLC2A1,CA1,SPTB |
| | Monarch HPO | HP:0001892 | Abnormal bleeding | 5 | 411 | 1.01 | 0.0133 | CAT,FGB,FGA,FGG,FN1 |
| | Monarch HPO | HP:0000421 | Epistaxis | 3 | 78 | 1.51 | 0.0144 | FGB,FGA,FGG |
| | Monarch HPO | EFO:0005192 | Red blood cell distribution width | 6 | 713 | 0.85 | 0.017 | SLC4A1,OXSR1,HBB,HBD,SLC2A1,SPTB |
| | Monarch HPO | HP:0001723 | Restrictive cardiomyopathy | 2 | 13 | 2.12 | 0.017 | SLC4A1,SPTB |
| | Monarch HPO | HP:0005502 | Increased red cell osmotic fragility | 2 | 13 | 2.12 | 0.017 | SLC4A1,SPTB |
| | Monarch HPO | HP:0025143 | Chills | 2 | 13 | 2.12 | 0.017 | SLC4A1,SPTB |
| | Monarch HPO | HP:0001927 | Acanthocytosis | 2 | 14 | 2.08 | 0.0185 | SLC4A1,SPTB |
| | Monarch HPO | HP:0025548 | Increased mean corpuscular hemoglobin concentration | 2 | 14 | 2.08 | 0.0185 | SLC4A1,SPTB |
| | Monarch HPO | HP:0000707 | Abnormality of the nervous system | 12 | 3470 | 0.47 | 0.0214 | CAT,SLC4A1,FGB,FGA,HBB,FGG,FN1,PNP,ALAD,SLC2A1,LDHA,SPTB |
| | Monarch HPO | HP:0040186 | Maculopapular exanthema | 2 | 16 | 2.03 | 0.0225 | SLC4A1,SPTB |
| | Monarch HPO | HP:0003270 | Abdominal distention | 3 | 100 | 1.41 | 0.0252 | SLC4A1,ALAD,SPTB |
| | Monarch HPO | HP:0200042 | Skin ulcer | 3 | 100 | 1.41 | 0.0252 | SLC4A1,HBB,SPTB |
| | Monarch HPO | HP:0001997 | Gout | 2 | 18 | 1.98 | 0.0261 | SLC4A1,SPTB |
| | Monarch HPO | HP:0000988 | Skin rash | 3 | 103 | 1.39 | 0.0263 | SLC4A1,FGA,SPTB |
| | Monarch HPO | HP:0000124 | Renal tubular dysfunction | 3 | 109 | 1.37 | 0.0303 | SLC4A1,HBB,FN1 |
| | Monarch HPO | HP:0002239 | Gastrointestinal hemorrhage | 3 | 111 | 1.36 | 0.0316 | FGB,FGA,FGG |
| | Monarch HPO | EFO:0007986 | Reticulocyte count | 5 | 527 | 0.91 | 0.0326 | SLC4A1,BLVRB,ALAD,CA1,SPTB |
| | Monarch HPO | HP:0011842 | Abnormal skeletal morphology | 10 | 2573 | 0.52 | 0.0344 | SLC4A1,FGB,FGA,HBB,FGG,FN1,PNP,ALAD,SLC2A1,SPTB |
| | Monarch HPO | HP:0000790 | Hematuria | 3 | 118 | 1.34 | 0.0365 | FGA,HBB,FN1 |
| | Monarch HPO | HP:0002240 | Hepatomegaly | 5 | 544 | 0.89 | 0.0365 | SLC4A1,FGA,HBB,SLC2A1,SPTB |
| | Monarch HPO | EFO:0004509 | Hemoglobin measurement | 7 | 1228 | 0.69 | 0.0372 | SLC4A1,OXSR1,HBB,HBD,SLC2A1,CA1,SPTB |
| | Monarch HPO | HP:0003470 | Paralysis | 3 | 122 | 1.32 | 0.0383 | SLC4A1,ALAD,SLC2A1 |
| | Monarch HPO | EFO:0004526 | Mean corpuscular volume | 6 | 872 | 0.77 | 0.0387 | SLC4A1,OXSR1,HBB,SLC2A1,CA1,SPTB |
| | Monarch HPO | HP:0003265 | Neonatal hyperbilirubinemia | 2 | 24 | 1.85 | 0.0387 | SLC4A1,SPTB |
| | Monarch HPO | HP:0003326 | Myalgia | 3 | 127 | 1.3 | 0.0414 | SLC4A1,LDHA,SPTB |
| | Monarch HPO | HP:0000118 | Phenotypic abnormality | 14 | 5134 | 0.37 | 0.0437 | CAT,SLC4A1,FGB,FGA,HBB,FGG,FN1,PNP,C1QC,HBD,ALAD,SLC2A1,LDHA,SPTB |
| | Monarch HPO | HP:0002725 | Systemic lupus erythematosus | 2 | 26 | 1.82 | 0.0437 | PNP,C1QC |
| | Monarch HPO | HP:0001433 | Hepatosplenomegaly | 3 | 136 | 1.27 | 0.0481 | SLC4A1,HBB,SLC2A1 |
| | DISEASES | DOID:589 | Congenital hemolytic anemia | 6 | 55 | 1.97 | 3.00E-07 | HBA2,SLC4A1,HBB,HBD,SLC2A1,SPTB |
| | DISEASES | DOID:74 | Hematopoietic system disease | 9 | 409 | 1.27 | 5.76E-07 | HBA2,SLC4A1,FGB,FGA,HBB,FGG,HBD,SLC2A1,SPTB |
| | DISEASES | DOID:2236 | Congenital afibrinogenemia | 3 | 3 | 2.93 | 2.05E-05 | FGB,FGA,FGG |
| | DISEASES | DOID:10241 | Thalassemia | 3 | 14 | 2.26 | 0.00059 | HBA2,HBB,HBD |
| | DISEASES | DOID:5378 | Hemoglobin d disease | 2 | 2 | 2.93 | 0.0043 | HBB,HBD |

| | | | | | | | | |
|---|---|---|---|---|---|---|---|---|
| | DISEASES | DOID:4 | Disease | 17 | 5921 | 0.39 | 0.0099 | CAT,HBA2,SLC4A1,FGB,FGA,HBB,FGG,FN1,PNP,TGM2,C1QC,HBD,ALAD,SLC2A1,CA1,LDHA,SPTB |
| | DISEASES | DOID:0110031 | Hemoglobin h disease | 2 | 5 | 2.53 | 0.0121 | HBA2,HBB |
| | DISEASES | DOID:12241 | Beta thalassemia | 2 | 6 | 2.45 | 0.0146 | HBB,HBD |
| | DISEASES | DOID:2373 | Hereditary elliptocytosis | 2 | 8 | 2.33 | 0.0215 | SLC4A1,SPTB |
| | DISEASES | DOID:9120 | Amyloidosis | 3 | 70 | 1.56 | 0.026 | FGA,HBB,FN1 |
| | DISEASES | DOID:0050737 | Autosomal recessive disease | 8 | 1422 | 0.68 | 0.0352 | HBA2,FGB,FGA,HBB,FGG,PNP,TGM2,HBD |
| | DISEASES | DOID:12971 | Hereditary spherocytosis | 2 | 13 | 2.12 | 0.0352 | SLC4A1,SPTB |
| RED | TISSUES | BTO:0000089 | Blood | 19 | 1675 | 0.98 | 6.93E-14 | CAT,HBA2,THBS1,SLC4A1,BLVRB,PRDX2,FGB,FGA,HBB,FGG,PRDX6,FN1,PNP,C1QC,HBD,ALAD,CA1,LDHA,SPTB |
| | TISSUES | BTO:0000570 | Hematopoietic system | 20 | 2543 | 0.83 | 2.30E-12 | CAT,HBA2,THBS1,SLC4A1,BLVRB,PRDX2,FGB,FGA,HBB,FGG,PRDX6,FN1,PNP,TGM2,C1QC,HBD,ALAD,CA1,LDHA,SPTB |
| | TISSUES | BTO:0000759 | Liver | 18 | 1882 | 0.91 | 7.11E-12 | CAT,HBA2,THBS1,SLC4A1,BLVRB,PRDX2,FGB,FGA,HBB,FGG,PRDX6,FN1,PNP,TGM2,ALAD,SLC2A1,LDHA,SPTB |
| | TISSUES | BTO:0000345 | Digestive gland | 19 | 2645 | 0.79 | 7.92E-11 | CAT,HBA2,THBS1,SLC4A1,BLVRB,PRDX2,FGB,FGA,HBB,FGG,PRDX6,FN1,PNP,TGM2,ALAD,SLC2A1,CA1,LDHA,SPTB |
| BLUE | TISSUES | BTO:0000132 | Blood platelet | 10 | 337 | 1.4 | 9.49E-10 | CAT,HBA2,THBS1,FGB,FGA,FGG,PRDX6,PNP,ALAD,SPTB |
| GREEN | TISSUES | BTO:0000424 | Erythrocyte | 5 | 39 | 2.04 | 4.02E-07 | CAT,BLVRB,PRDX2,HBB,PRDX6 |
| | TISSUES | BTO:0001486 | Skeletal system | 12 | 1203 | 0.93 | 5.22E-07 | HBA2,THBS1,FGB,FGA,HBB,FGG,PRDX6,FN1,TGM2,SLC2A1,LDHA,SPTB |
| | TISSUES | BTO:0001491 | Viscus | 19 | 5020 | 0.51 | 3.74E-06 | CAT,HBA2,THBS1,SLC4A1,BLVRB,PRDX2,FGB,FGA,HBB,FGG,PRDX6,FN1,PNP,TGM2,ALAD,SLC2A1,CA1,LDHA,SPTB |
| | TISSUES | BTO:0000574 | Hematopoietic cell | 10 | 933 | 0.96 | 7.83E-06 | CAT,SLC4A1,BLVRB,PRDX2,HBB,PRDX6,FN1,C1QC,HBD,LDHA |
| | TISSUES | BTO:0000562 | Heart | 7 | 695 | 0.93 | 0.0016 | THBS1,FGA,HBB,FN1,TGM2,LDHA,SPTB |
| | TISSUES | BTO:0000284 | Organism form | 11 | 2239 | 0.62 | 0.0022 | CAT,HBA2,THBS1,PSMB1,OXSR1,HBB,FN1,TGM2,ALAD,SLC2A1,LDHA |
| | TISSUES | BTO:0001489 | Whole body | 23 | 12619 | 0.19 | 0.0053 | CAT,HBA2,THBS1,PSMB1,SLC4A1,BLVRB,PRDX2,FGB,FGA,OXSR1,HBB,FGG,PRDX6,FN1,PNP,TGM2,C1QC,HBD,ALAD,SLC2A1,CA1,LDHA,SPTB |
| | TISSUES | BTO:0000174 | Embryonic structure | 10 | 2132 | 0.6 | 0.0085 | CAT,HBA2,THBS1,PSMB1,OXSR1,HBB,FN1,TGM2,SLC2A1,LDHA |
| | TISSUES | BTO:0000203 | Respiratory system | 8 | 1436 | 0.68 | 0.0163 | HBA2,THBS1,PSMB1,PRDX2,FN1,TGM2,SLC2A1,LDHA |
| | TISSUES | BTO:0000141 | Bone marrow | 5 | 478 | 0.95 | 0.02 | THBS1,FGA,PRDX6,FN1,LDHA |
| | TISSUES | BTO:0001090 | Mouth | 5 | 482 | 0.95 | 0.02 | HBA2,FN1,PNP,ALAD,SLC2A1 |
| | TISSUES | BTO:0002731 | Erythroid cell | 2 | 16 | 2.03 | 0.02 | SLC4A1,HBD |
| | TISSUES | BTO:0000763 | Lung | 7 | 1162 | 0.71 | 0.0241 | HBA2,THBS1,PSMB1,PRDX2,FN1,TGM2,LDHA |
| | TISSUES | BTO:0001078 | Placenta | 7 | 1176 | 0.7 | 0.0248 | HBA2,THBS1,HBB,FN1,TGM2,SLC2A1,LDHA |
| | TISSUES | BTO:0000421 | Connective tissue | 6 | 871 | 0.77 | 0.0316 | CAT,BLVRB,PRDX2,PRDX6,FN1,SLC2A1 |
| | TISSUES | BTO:0001176 | Endothelial cell | 3 | 133 | 1.28 | 0.0383 | THBS1,FN1,TGM2 |
| | TISSUES | BTO:0000394 | Aorta endothelium | 2 | 32 | 1.73 | 0.0492 | THBS1,FN1 |
| YELLOW | COMPARTMENTS | GOCC:0072562 | Blood microparticle | 10 | 116 | 1.87 | 2.07E-13 | HBA2,SLC4A1,FGB,FGA,HBB,FGG,FN1,C1QC,HBD,SLC2A1 |
| | COMPARTMENTS | GOCC:0005615 | Extracellular space | 13 | 985 | 1.05 | 1.18E-08 | HBA2,THBS1,SLC4A1,FGB,FGA,HBB,FGG,FN1,TGM2,C1QC,HBD,SLC2A1,LDHA |
| VIOLET | COMPARTMENTS | GOCC:0005577 | Fibrinogen complex | 5 | 19 | 2.35 | 4.52E-08 | THBS1,FGB,FGA,FGG,FN1 |
| | COMPARTMENTS | GOCC:0030141 | Secretory granule | 11 | 704 | 1.12 | 7.43E-08 | CAT,THBS1,PSMB1,FGB,FGA,HBB,FGG,PRDX6,FN1,PNP,ALAD |
| | COMPARTMENTS | GOCC:0031982 | Vesicle | 15 | 2051 | 0.79 | 1.86E-07 | CAT,HBA2,THBS1,PSMB1,FGB,FGA,HBB,FGG,PRDX6,FN1,PNP,TGM2,ALAD,SLC2A1,LDHA |

| | | | | | | | | |
|---|---|---|---|---|---|---|---|---|
| | COMPARTMENTS | GOCC:0031093 | Platelet alpha granule lumen | 5 | 68 | 1.8 | 6.59E-06 | THBS1,FGB,FGA,FGG,FN1 |
| | COMPARTMENTS | GOCC:0005737 | Cytoplasm | 22 | 7871 | 0.38 | 8.29E-06 | CAT,HBA2,THBS1,PSMB1,SLC4A1,BLVRB,PRDX2,FGB,FGA,OXSR1,HBB,FGG,PRDX6,FN1,PNP,TGM2,HBD,ALAD,SLC2A1,CA1,LDHA,SPTB |
| | COMPARTMENTS | GOCC:0031410 | Cytoplasmic vesicle | 12 | 1709 | 0.78 | 2.31E-05 | CAT,HBA2,THBS1,PSMB1,FGB,FGA,HBB,FGG,PRDX6,FN1,PNP,ALAD |
| | COMPARTMENTS | GOCC:0034774 | Secretory granule lumen | 6 | 230 | 1.35 | 4.34E-05 | THBS1,FGB,FGA,FGG,PRDX6,FN1 |
| | COMPARTMENTS | GOCC:1904813 | ficolin-1-rich granule lumen | 5 | 125 | 1.53 | 5.26E-05 | CAT,PSMB1,HBB,PNP,ALAD |
| | COMPARTMENTS | GOCC:0031838 | Haptoglobin-hemoglobin complex | 3 | 11 | 2.37 | 6.55E-05 | HBA2,HBB,HBD |
| | COMPARTMENTS | GOCC:0005833 | Hemoglobin complex | 3 | 22 | 2.06 | 0.00039 | HBA2,HBB,HBD |
| | COMPARTMENTS | GOCC:0005829 | Cytosol | 13 | 2919 | 0.58 | 0.00055 | CAT,HBA2,PSMB1,BLVRB,OXSR1,HBB,PRDX6,PNP,TGM2,HBD,CA1,LDHA,SPTB |
| | COMPARTMENTS | GOCC:0009897 | External side of plasma membrane | 4 | 104 | 1.51 | 0.00079 | THBS1,FGB,FGA,FGG |
| | COMPARTMENTS | GOCC:1903561 | Extracellular vesicle | 6 | 444 | 1.06 | 0.0011 | THBS1,HBB,FN1,TGM2,SLC2A1,LDHA |
| | COMPARTMENTS | GOCC:0098552 | Side of membrane | 5 | 266 | 1.2 | 0.0013 | THBS1,FGB,FGA,FGG,SPTB |
| | COMPARTMENTS | GOCC:0070013 | Intracellular organelle lumen | 12 | 2812 | 0.56 | 0.0021 | CAT,HBA2,THBS1,PSMB1,FGB,FGA,HBB,FGG,PRDX6,FN1,PNP,ALAD |
| | COMPARTMENTS | GOCC:0005788 | Endoplasmic reticulum lumen | 4 | 150 | 1.36 | 0.0024 | THBS1,FGA,FGG,FN1 |
| | COMPARTMENTS | GOCC:0070062 | Extracellular exosome | 5 | 368 | 1.06 | 0.0048 | THBS1,FN1,TGM2,SLC2A1,LDHA |
| | COMPARTMENTS | GOCC:0030863 | Cortical cytoskeleton | 3 | 71 | 1.56 | 0.0065 | SLC4A1,SLC2A1,SPTB |
| | COMPARTMENTS | GOCC:0009986 | Cell surface | 5 | 433 | 0.99 | 0.0095 | THBS1,FGB,FGA,FGG,SPTB |
| | COMPARTMENTS | GOCC:0071682 | Endocytic vesicle lumen | 2 | 22 | 1.89 | 0.0243 | HBA2,HBB |
| | COMPARTMENTS | GOCC:0110165 | Cellular anatomical entity | 23 | 13853 | 0.15 | 0.0243 | CAT,HBA2,THBS1,PSMB1,SLC4A1,BLVRB,PRDX2,FGB,FGA,OXSR1,HBB,FGG,PRDX6,FN1,PNP,TGM2,C1QC,HBD,ALAD,SLC2A1,CA1,LDHA,SPTB |
| | UniProt Keywords | KW-0360 | Hereditary hemolytic anemia | 5 | 33 | 2.11 | 4.63E-07 | HBA2,SLC4A1,HBB,SLC2A1,SPTB |
| | UniProt Keywords | KW-0561 | Oxygen transport | 3 | 11 | 2.37 | 0.00017 | HBA2,HBB,HBD |
| | UniProt Keywords | KW-0007 | Acetylation | 14 | 3341 | 0.55 | 0.00071 | CAT,HBA2,PSMB1,SLC4A1,PRDX2,OXSR1,HBB,PRDX6,PNP,TGM2,HBD,SLC2A1,CA1,LDHA |
| | UniProt Keywords | KW-0575 | Peroxidase | 3 | 26 | 1.99 | 0.00086 | CAT,PRDX2,PRDX6 |
| | UniProt Keywords | KW-0225 | Disease mutation | 13 | 3145 | 0.55 | 0.0015 | HBA2,SLC4A1,FGB,FGA,HBB,FGG,FN1,PNP,C1QC,ALAD,SLC2A1,LDHA,SPTB |
| | UniProt Keywords | KW-0349 | Heme | 4 | 121 | 1.45 | 0.0015 | CAT,HBA2,HBB,HBD |
| | UniProt Keywords | KW-0094 | Blood coagulation | 3 | 46 | 1.74 | 0.0024 | FGB,FGA,FGG |
| | UniProt Keywords | KW-0250 | Elliptocytosis | 2 | 8 | 2.33 | 0.0044 | SLC4A1,SPTB |
| | UniProt Keywords | KW-0597 | Phosphoprotein | 19 | 8057 | 0.3 | 0.0044 | CAT,HBA2,PSMB1,SLC4A1,BLVRB,PRDX2,FGA,OXSR1,HBB,FGG,PRDX6,FN1,PNP,TGM2,HBD,ALAD,SLC2A1,LDHA,SPTB |
| | UniProt Keywords | KW-0971 | Glycation | 2 | 12 | 2.15 | 0.0073 | HBA2,HBB |

| | | | | | | | | |
|---|---|---|---|---|---|---|---|---|
| | UniProt Keywords | KW-0049 | Antioxidant | 2 | 14 | 2.08 | 0.0087 | PRDX2,PRDX6 |
| | UniProt Keywords | KW-0560 | Oxidoreductase | 5 | 568 | 0.87 | 0.0207 | CAT,BLVRB,PRDX2,PRDX6,LDHA |
| | Pfam | PF08702 | Fibrinogen alpha/beta chain family | 3 | 3 | 2.93 | 9.72E-05 | FGB,FGA,FGG |
| | Pfam | PF00042 | Globin | 3 | 13 | 2.29 | 0.0014 | HBA2,HBB,HBD |
| | Pfam | PF00147 | Fibrinogen beta and gamma chains, C-terminal globular domain | 3 | 24 | 2.03 | 0.0047 | FGB,FGA,FGG |
| | Pfam | PF10417 | C-terminal domain of 1-Cys peroxiredoxin | 2 | 5 | 2.53 | 0.0238 | PRDX2,PRDX6 |
| | Pfam | PF08534 | Redoxin | 2 | 6 | 2.45 | 0.0253 | PRDX2,PRDX6 |
| | InterPro | IPR012290 | Fibrinogen, alpha/beta/gamma chain, coiled coil domain | 3 | 3 | 2.93 | 0.00019 | FGB,FGA,FGG |
| | InterPro | IPR000971 | Globin | 3 | 11 | 2.37 | 0.0017 | HBA2,HBB,HBD |
| | InterPro | IPR009050 | Globin-like superfamily | 3 | 11 | 2.37 | 0.0017 | HBA2,HBB,HBD |
| | InterPro | IPR012292 | Globin/Protoglobin | 3 | 11 | 2.37 | 0.0017 | HBA2,HBB,HBD |
| | InterPro | IPR020837 | Fibrinogen, conserved site | 3 | 19 | 2.13 | 0.0029 | FGB,FGA,FGG |
| | InterPro | IPR014716 | Fibrinogen, alpha/beta/gamma chain, C-terminal globular, subdomain 1 | 3 | 26 | 1.99 | 0.0057 | FGB,FGA,FGG |
| | InterPro | IPR002181 | Fibrinogen, alpha/beta/gamma chain, C-terminal globular domain | 3 | 32 | 1.9 | 0.0087 | FGB,FGA,FGG |
| | InterPro | IPR036056 | Fibrinogen-like, C-terminal | 3 | 32 | 1.9 | 0.0087 | FGB,FGA,FGG |
| | InterPro | IPR024706 | Peroxiredoxin, AhpC-type | 2 | 3 | 2.75 | 0.0099 | PRDX2,PRDX6 |
| | InterPro | IPR002337 | Haemoglobin, beta-type | 2 | 4 | 2.63 | 0.0134 | HBB,HBD |
| | InterPro | IPR000866 | Alkyl hydroperoxide reductase subunit C/ Thiol specific antioxidant | 2 | 5 | 2.53 | 0.017 | PRDX2,PRDX6 |
| | InterPro | IPR019479 | Peroxiredoxin, C-terminal | 2 | 5 | 2.53 | 0.017 | PRDX2,PRDX6 |
| | SMART | SM01212 | Fibrinogen alpha/beta chain family | 3 | 3 | 2.93 | 2.05E-05 | FGB,FGA,FGG |
| | SMART | SM00186 | Fibrinogen-related domains (FReDs) | 3 | 27 | 1.98 | 0.002 | FGB,FGA,FGG |

**Supplementary table 2.** STRING protein interaction and functional enrichment analysis of the 23 proteins spatially most closely regulated with the respect to solute carrier family 2 (GTR1) [3]. The red color highlights proteins linked by the "BTO:0000089 Blood" (19 partners, FDR = 6.93e-14) term. Proteins highlighted in blue are linked by the "BTO:0000132 Blood platelet" (10 partners, FDR = 9.49e-10) term. The green color highlights protein partners linked by the "BTO:0000424 Erythrocyte" (5 partners, FDR = 4.02e-07) term. The Yellow and violet colored hits were found in the Subcellular localization (compartment) analysis which linked them via the "Blood microparticle" (10 partners, FDR = 2.07e-13) and the Fibrinogen complex (5 partners, FDR = 4.52e-08) terms. Dark green colored hits were found in the Cellular Component (Gene Ontology) analysis which linked them by the “Blood microparticle” (10 partners, FDR = 9.67e-14) term. Biochemical processes and pathways linked the 23 queries by the “Blood coagulation” (6 partners, FDR = 0.00036, cyan), “GO:0042060_WoundHealing” (7 partners, FDR = 0.00020, gold), GO:0042744_H.PeroxideCatabolicProcess (6 partners, FDR = 2.18e-08, dark violet) GO terms. While, the KEGG pathway analysis revealed enrichment of the “hsa04610_Platelet_Activation” (3 partners, FDR = 0.0458, brown) and the "Complement and coagulation cascades" (4 partners, FDR = 0.00097, grey) terms.

| Protein | PIP_HUMAN adjacency values | Protein | PIP_HUMAN adjacency values | Protein | PIP_HUMAN adjacency values | Protein | PIP_HUMAN adjacency values |
|---|---|---|---|---|---|---|---|
| ENOA_HUMAN | 1.11E-08 | MYH9_HUMAN | 6.74E-06 | CAN1_HUMAN | 5.00E-05 | FBN1_HUMAN | 2.16E-04 |
| HSP7C_HUMAN | 1.66E-08 | NONO_HUMAN | 6.78E-06 | KAP0_HUMAN | 5.03E-05 | 1433E_HUMAN | 2.18E-04 |
| H10_HUMAN | 1.44E-07 | COGA1_HUMAN | 6.88E-06 | CSRP1_HUMAN | 5.24E-05 | GLU2B_HUMAN | 2.20E-04 |
| ABHEB_HUMAN | 1.51E-07 | CLH1_HUMAN | 6.91E-06 | LAMA5_HUMAN | 5.28E-05 | FCGBP_HUMAN | 2.21E-04 |
| KPYM_HUMAN | 1.81E-07 | ANXA4_HUMAN | 7.23E-06 | HNRPD_HUMAN | 5.37E-05 | RISC_HUMAN | 2.26E-04 |
| HNRPC_HUMAN | 2.63E-07 | H1X_HUMAN | 7.98E-06 | PDIA1_HUMAN | 5.45E-05 | CAN2_HUMAN | 2.29E-04 |
| OST48_HUMAN | 2.70E-07 | COFA1_HUMAN | 8.22E-06 | DUS3L_HUMAN | 5.46E-05 | FSCN1_HUMAN | 2.43E-04 |
| PRDX3_HUMAN | 3.00E-07 | PGK1_HUMAN | 8.51E-06 | HP1B3_HUMAN | 5.69E-05 | LAMB1_HUMAN | 2.57E-04 |
| BIP_HUMAN | 3.31E-07 | H4_HUMAN | 8.74E-06 | AL7A1_HUMAN | 5.87E-05 | ODPB_HUMAN | 2.69E-04 |
| CH60_HUMAN | 4.34E-07 | XRCC6_HUMAN | 8.98E-06 | CATD_HUMAN | 6.00E-05 | PCBP1_HUMAN | 3.05E-04 |
| PLEC_HUMAN | 6.02E-07 | ATPO_HUMAN | 9.64E-06 | MFAP4_HUMAN | 6.19E-05 | CAC1A_HUMAN | 3.31E-04 |
| CNDP2_HUMAN | 7.24E-07 | IQGA1_HUMAN | 9.82E-06 | SERA_HUMAN | 6.24E-05 | TMM43_HUMAN | 3.55E-04 |
| LRC47_HUMAN | 8.68E-07 | SSRD_HUMAN | 1.06E-05 | ROA3_HUMAN | 6.81E-05 | HLAA_HUMAN | 3.56E-04 |
| RL7_HUMAN | 8.98E-07 | AHNK_HUMAN | 1.15E-05 | DNPEP_HUMAN | 6.91E-05 | RPN1_HUMAN | 3.78E-04 |
| EFTU_HUMAN | 9.10E-07 | PRDX1_HUMAN | 1.30E-05 | RL6_HUMAN | 7.34E-05 | **K2C5_HUMAN** | 3.79E-04 |
| AK1A1_HUMAN | 9.51E-07 | CAPG_HUMAN | 1.40E-05 | **MOES_HUMAN** | 7.80E-05 | ACADV_HUMAN | 3.93E-04 |
| ACTN4_HUMAN | 1.09E-06 | GPX3_HUMAN | 1.44E-05 | RRBP1_HUMAN | 7.83E-05 | RBP2_HUMAN | 3.98E-04 |
| NUCL_HUMAN | 1.30E-06 | TCP4_HUMAN | 1.59E-05 | UBA1_HUMAN | 8.16E-05 | FLNA_HUMAN | 4.16E-04 |
| LASP1_HUMAN | 1.32E-06 | DYHC1_HUMAN | 1.68E-05 | CO4A2_HUMAN | 8.50E-05 | ENPL_HUMAN | 4.35E-04 |
| HS71A_HUMAN | 1.46E-06 | ATPB_HUMAN | 1.78E-05 | PRAF3_HUMAN | 8.53E-05 | DHE3_HUMAN | 4.65E-04 |
| NPM_HUMAN | 1.67E-06 | PDCD6_HUMAN | 1.82E-05 | KREM2_HUMAN | 9.19E-05 | CDC42_HUMAN | 4.71E-04 |
| MDHM_HUMAN | 1.67E-06 | RL8_HUMAN | 1.87E-05 | **K1C19_HUMAN** | 9.59E-05 | RL10A_HUMAN | 5.17E-04 |
| RL31_HUMAN | 1.76E-06 | CBR1_HUMAN | 2.09E-05 | GRP75_HUMAN | 9.73E-05 | CPNS1_HUMAN | 5.19E-04 |
| RL12_HUMAN | 1.81E-06 | PDIA6_HUMAN | 2.11E-05 | TBB5_HUMAN | 9.93E-05 | STT3B_HUMAN | 5.55E-04 |
| HS90A_HUMAN | 1.83E-06 | CH10_HUMAN | 2.39E-05 | PTBP1_HUMAN | 1.07E-04 | NCK5L_HUMAN | 5.56E-04 |
| FBLN5_HUMAN | 1.88E-06 | IF4H_HUMAN | 2.48E-05 | HCD2_HUMAN | 1.19E-04 | COIA1_HUMAN | 5.69E-04 |
| EF2_HUMAN | 1.93E-06 | SEPT2_HUMAN | 2.61E-05 | HCDH_HUMAN | 1.20E-04 | ARK72_HUMAN | 5.96E-04 |
| TINAL_HUMAN | 2.01E-06 | CO7A1_HUMAN | 2.90E-05 | PHB1_HUMAN | 1.27E-04 | | |
| PYGB_HUMAN | 2.02E-06 | HNRPK_HUMAN | 2.93E-05 | RAB7A_HUMAN | 1.29E-04 | ANXA7_HUMAN | 6.79E-04 |
| H2AY_HUMAN | 2.14E-06 | RS18_HUMAN | 2.96E-05 | PGBM_HUMAN | 1.31E-04 | PEF1_HUMAN | 7.06E-04 |
| PHB2_HUMAN | 2.17E-06 | TERA_HUMAN | 3.10E-05 | LMNA_HUMAN | 1.32E-04 | COF1_HUMAN | 7.71E-04 |
| LMNB2_HUMAN | 2.25E-06 | RL13_HUMAN | 3.12E-05 | COX2_HUMAN | 1.33E-04 | IDHP_HUMAN | 8.24E-04 |
| MYL6_HUMAN | 2.54E-06 | BCAM_HUMAN | 3.13E-05 | ANXA1_HUMAN | 1.36E-04 | RS15_HUMAN | 8.55E-04 |
| ROA2_HUMAN | 2.72E-06 | FRIL_HUMAN | 3.35E-05 | SND1_HUMAN | 1.40E-04 | GDIR1_HUMAN | 9.89E-04 |
| EMIL1_HUMAN | 2.93E-06 | HNRH3_HUMAN | 3.37E-05 | ACTN1_HUMAN | 1.43E-04 | TAGL2_HUMAN | 1.01E-03 |
| CAP1_HUMAN | 3.68E-06 | PEBP1_HUMAN | 3.46E-05 | TPIS_HUMAN | 1.47E-04 | RS28_HUMAN | 1.04E-03 |
| PDIA3_HUMAN | 3.78E-06 | CO4A1_HUMAN | 3.52E-05 | H2A2B_HUMAN | 1.54E-04 | RTN4_HUMAN | 1.07E-03 |
| WDR1_HUMAN | 3.93E-06 | DPYL3_HUMAN | 3.77E-05 | CNN1_HUMAN | 1.65E-04 | COR1B_HUMAN | 1.07E-03 |
| G3P_HUMAN | 4.14E-06 | ANXA3_HUMAN | 4.08E-05 | PUR9_HUMAN | 1.65E-04 | CISY_HUMAN | 1.13E-03 |
| PRDX5_HUMAN | 4.29E-06 | TXND5_HUMAN | 4.23E-05 | LSM3_HUMAN | 1.74E-04 | TM109_HUMAN | 1.22E-03 |
| **K1C18_HUMAN** | 4.30E-06 | PLAK_HUMAN | 4.46E-05 | RD23B_HUMAN | 1.88E-04 | RSSA_HUMAN | 1.25E-03 |
| PPIA_HUMAN | 4.47E-06 | RHG01_HUMAN | 4.48E-05 | SRSF3_HUMAN | 1.93E-04 | SC22B_HUMAN | 1.28E-03 |
| HNRPM_HUMAN | 5.19E-06 | RS16_HUMAN | 4.64E-05 | SFPQ_HUMAN | 1.93E-04 | SPB6_HUMAN | 1.28E-03 |
| HNRPU_HUMAN | 5.99E-06 | MYH11_HUMAN | 4.90E-05 | GANAB_HUMAN | 2.01E-04 | SPTN1_HUMAN | 1.28E-03 |
| HS90B_HUMAN | 6.13E-06 | RS24_HUMAN | 4.98E-05 | SODM_HUMAN | 2.13E-04 | NID2_HUMAN | 1.33E-03 |

| Protein | PIP_HUMAN adjacency values | Protein | PIP_HUMAN adjacency values | Protein | PIP_HUMAN adjacency values | Protein | PIP_HUMAN adjacency values |
|---|---|---|---|---|---|---|---|
| TKT_HUMAN | 1.36E-03 | S20A2_HUMAN | 5.32E-03 | PNCB_HUMAN | 1.95E-02 | CO6A2_HUMAN | 4.86E-02 |
| RINI_HUMAN | 1.38E-03 | ANXA2_HUMAN | 5.32E-03 | CO5A1_HUMAN | 1.98E-02 | G6PI_HUMAN | 4.89E-02 |
| RACK1_HUMAN | 1.42E-03 | ACOHC_HUMAN | 5.58E-03 | CT144_HUMAN | 2.00E-02 | AACT_HUMAN | 5.00E-02 |
| CO5A2_HUMAN | 1.43E-03 | MPCP_HUMAN | 5.70E-03 | HSPB1_HUMAN | 2.22E-02 | SRPX_HUMAN | 5.07E-02 |
| TENX_HUMAN | 1.56E-03 | TCPE_HUMAN | 6.12E-03 | CUTA_HUMAN | 2.22E-02 | HPT_HUMAN | 5.17E-02 |
| GSTP1_HUMAN | 1.60E-03 | THRB_HUMAN | 6.15E-03 | IF5A1_HUMAN | 2.27E-02 | PRDX2_HUMAN | 5.29E-02 |
| SPTB2_HUMAN | 1.60E-03 | CROCC_HUMAN | 6.25E-03 | CFAB_HUMAN | 2.37E-02 | CERU_HUMAN | 5.38E-02 |
| TLN1_HUMAN | 1.61E-03 | NB5R3_HUMAN | 6.57E-03 | PCOC1_HUMAN | 2.43E-02 | NOS1_HUMAN | 5.41E-02 |
| 1433Z_HUMAN | 1.79E-03 | PRR12_HUMAN | 7.25E-03 | RT09_HUMAN | 2.47E-02 | IGHM_HUMAN | 5.42E-02 |
| MZB1_HUMAN | 1.80E-03 | PCYOX_HUMAN | 7.35E-03 | CO1A2_HUMAN | 2.48E-02 | IGJ_HUMAN | 5.43E-02 |
| TPP1_HUMAN | 1.87E-03 | TETN_HUMAN | 7.53E-03 | EHD2_HUMAN | 2.65E-02 | KCD12_HUMAN | 5.48E-02 |
| DPOLQ_HUMAN | 1.89E-03 | BGH3_HUMAN | 7.89E-03 | FBLN1_HUMAN | 2.79E-02 | HEM2_HUMAN | 5.58E-02 |
| TAGL_HUMAN | 2.05E-03 | PA1B2_HUMAN | 7.93E-03 | IPSP_HUMAN | 2.80E-02 | OLFL3_HUMAN | 5.58E-02 |
| VAT1_HUMAN | 2.09E-03 | PGM1_HUMAN | 8.06E-03 | IGHA1_HUMAN | 2.86E-02 | ZA2G_HUMAN | 5.63E-02 |
| PARK7_HUMAN | 2.13E-03 | 6PGD_HUMAN | 8.16E-03 | GDIB_HUMAN | 2.98E-02 | COCA1_HUMAN | 5.69E-02 |
| LAMC1_HUMAN | 2.44E-03 | ARPC4_HUMAN | 8.59E-03 | CO8B_HUMAN | 2.99E-02 | TLN2_HUMAN | 5.77E-02 |
| TALDO_HUMAN | 2.45E-03 | COEA1_HUMAN | 8.69E-03 | PSME2_HUMAN | 3.06E-02 | A1BG_HUMAN | 5.78E-02 |
| ALDOA_HUMAN | 2.45E-03 | ILEU_HUMAN | 8.94E-03 | RS25_HUMAN | 3.11E-02 | UGPA_HUMAN | 5.84E-02 |
| MYL9_HUMAN | 2.45E-03 | 2AAA_HUMAN | 9.12E-03 | ALDH2_HUMAN | 3.12E-02 | ANXA5_HUMAN | 5.90E-02 |
| AL1A1_HUMAN | 2.47E-03 | ESYT1_HUMAN | 9.17E-03 | MYO1C_HUMAN | 3.22E-02 | F13A_HUMAN | 6.04E-02 |
| PPIB_HUMAN | 2.61E-03 | POSTN_HUMAN | 9.46E-03 | SDHA_HUMAN | 3.27E-02 | CAVN1_HUMAN | 6.06E-02 |
| CSPG2_HUMAN | 2.78E-03 | PGRC1_HUMAN | 9.77E-03 | COLA1_HUMAN | 3.32E-02 | TSP1_HUMAN | 6.16E-02 |
| QCR1_HUMAN | 2.80E-03 | TRFL_HUMAN | 1.04E-02 | PRDX4_HUMAN | 3.33E-02 | SPTB1_HUMAN | 6.19E-02 |
| VINC_HUMAN | 2.83E-03 | CPNE3_HUMAN | 1.05E-02 | SAMP_HUMAN | 3.35E-02 | AEBP1_HUMAN | 6.20E-02 |
| EHD4_HUMAN | 2.88E-03 | DPYL2_HUMAN | 1.13E-02 | CAV1_HUMAN | 3.36E-02 | CO8G_HUMAN | 6.29E-02 |
| LZTS3_HUMAN | 2.90E-03 | ITB1_HUMAN | 1.14E-02 | A2GL_HUMAN | 3.41E-02 | RUBIC_HUMAN | 6.49E-02 |
| FBLN3_HUMAN | 3.00E-03 | GELS_HUMAN | 1.14E-02 | ANGT_HUMAN | 3.49E-02 | RRAS_HUMAN | 6.71E-02 |
| ECHB_HUMAN | 3.12E-03 | GGT5_HUMAN | 1.15E-02 | HYEP_HUMAN | 3.50E-02 | HEMO_HUMAN | 6.72E-02 |
| PLSL_HUMAN | 3.40E-03 | AL9A1_HUMAN | 1.16E-02 | HEP2_HUMAN | 3.53E-02 | ITIH4_HUMAN | 6.84E-02 |
| ALDOC_HUMAN | 3.40E-03 | PROF1_HUMAN | 1.17E-02 | CO9_HUMAN | 3.54E-02 | LBP_HUMAN | 7.37E-02 |
| GNAI2_HUMAN | 3.50E-03 | SYPL1_HUMAN | 1.18E-02 | IPYR_HUMAN | 3.58E-02 | IGHA2_HUMAN | 7.37E-02 |
| NID1_HUMAN | 3.57E-03 | PGRC2_HUMAN | 1.19E-02 | CO2A1_HUMAN | 3.67E-02 | ALBU_HUMAN | 7.38E-02 |
| TCPG_HUMAN | 3.83E-03 | ILK_HUMAN | 1.22E-02 | S10A6_HUMAN | 3.69E-02 | TRFE_HUMAN | 7.56E-02 |
| ATPA_HUMAN | 3.90E-03 | HRG_HUMAN | 1.29E-02 | THIL_HUMAN | 3.83E-02 | KVD33_HUMAN | 7.56E-02 |
| KAP2_HUMAN | 3.97E-03 | CO6A3_HUMAN | 1.41E-02 | PEDF_HUMAN | 3.87E-02 | PGS1_HUMAN | 7.68E-02 |
| SERPH_HUMAN | 4.04E-03 | ITIH1_HUMAN | 1.42E-02 | ITIH2_HUMAN | 3.99E-02 | PGRP2_HUMAN | 7.75E-02 |
| FUBP2_HUMAN | 4.12E-03 | ANX11_HUMAN | 1.46E-02 | PLIN4_HUMAN | 4.01E-02 | CRYAB_HUMAN | 7.93E-02 |
| CPNE1_HUMAN | 4.31E-03 | LAMB2_HUMAN | 1.48E-02 | MUC18_HUMAN | 4.09E-02 | DERM_HUMAN | 8.08E-02 |
| FGD6_HUMAN | 4.61E-03 | VTNC_HUMAN | 1.55E-02 | HV349_HUMAN | 4.20E-02 | LITD1_HUMAN | 8.15E-02 |
| DDAH2_HUMAN | 4.73E-03 | PSA_HUMAN | 1.59E-02 | OLFL1_HUMAN | 4.21E-02 | PLIN1_HUMAN | 8.24E-02 |
| SBP1_HUMAN | 5.11E-03 | CO1A1_HUMAN | 1.60E-02 | DPP4_HUMAN | 4.37E-02 | IC1_HUMAN | 8.31E-02 |
| THIO_HUMAN | 5.16E-03 | SH3L1_HUMAN | 1.61E-02 | RS9_HUMAN | 4.37E-02 | HV146_HUMAN | 8.38E-02 |
| FKB1A_HUMAN | 5.20E-03 | PSB2_HUMAN | 1.76E-02 | APOB_HUMAN | 4.48E-02 | ANXA6_HUMAN | 8.47E-02 |
| UBE2N_HUMAN | 5.20E-03 | IF172_HUMAN | 1.78E-02 | CO6A1_HUMAN | 4.54E-02 | GPDA_HUMAN | 8.52E-02 |
| VIME_HUMAN | 5.25E-03 | CO5A3_HUMAN | 1.82E-02 | AMBP_HUMAN | 4.80E-02 | FINC_HUMAN | 8.59E-02 |

| Protein | PIP_HUMAN adjacency values | Protein | PIP_HUMAN adjacency values | Protein | PIP_HUMAN adjacency values |
|---|---|---|---|---|---|
| CATA_HUMAN | 8.71E-02 | GTR1_HUMAN | 1.30E-01 | K1C10_HUMAN | 0.32 |
| CFAD_HUMAN | 8.73E-02 | PGS2_HUMAN | 1.34E-01 | HV374_HUMAN | 0.35 |
| LEG1_HUMAN | 8.80E-02 | PSA3_HUMAN | 1.35E-01 | CD44_HUMAN | 0.36 |
| APOA4_HUMAN | 8.90E-02 | ACSL1_HUMAN | 1.39E-01 | PSA1_HUMAN | 0.37 |
| LYSC_HUMAN | 8.92E-02 | LEG3_HUMAN | 0.14 | K1C9_HUMAN | 0.43 |
| AOC3_HUMAN | 8.94E-02 | CO3A1_HUMAN | 0.14 | KVD20_HUMAN | 0.43 |
| IGKC_HUMAN | 9.29E-02 | MIME_HUMAN | 0.15 | C1QC_HUMAN | 0.44 |
| B3AT_HUMAN | 9.36E-02 | FAAA_HUMAN | 0.15 | NDK3_HUMAN | 0.45 |
| FAS_HUMAN | 9.44E-02 | CAH1_HUMAN | 0.15 | HV226_HUMAN | 0.47 |
| CO6A6_HUMAN | 9.81E-02 | HBD_HUMAN | 0.15 | CYTB_HUMAN | 0.55 |
| ANT3_HUMAN | 9.82E-02 | SODE_HUMAN | 0.15 | K2C1_HUMAN | 0.55 |
| LAMA4_HUMAN | 9.86E-02 | APOA2_HUMAN | 0.17 | PIP_HUMAN | 1.00 |
| EST1_HUMAN | 9.88E-02 | HV338_HUMAN | 0.18 | | |
| CBPA3_HUMAN | 9.95E-02 | LDHB_HUMAN | 0.18 | | |
| FIBB_HUMAN | 9.96E-02 | ECM1_HUMAN | 0.19 | | |
| PRDX6_HUMAN | 1.01E-01 | FETUA_HUMAN | 0.19 | | |
| MDHC_HUMAN | 1.03E-01 | PLMN_HUMAN | 0.20 | | |
| HBB_HUMAN | 1.03E-01 | KV401_HUMAN | 0.20 | | |
| ASPN_HUMAN | 1.04E-01 | RMC1_HUMAN | 0.20 | | |
| CFAI_HUMAN | 1.04E-01 | LDHA_HUMAN | 0.21 | | |
| CO3_HUMAN | 1.04E-01 | HV307_HUMAN | 0.21 | | |
| APOA1_HUMAN | 1.05E-01 | PSB1_HUMAN | 0.21 | | |
| IGHG4_HUMAN | 1.05E-01 | A1AG1_HUMAN | 0.21 | | |
| PRELP_HUMAN | 1.06E-01 | LV861_HUMAN | 0.22 | | |
| KV320_HUMAN | 1.08E-01 | FABP4_HUMAN | 0.23 | | |
| VTDB_HUMAN | 1.09E-01 | IGHG1_HUMAN | 0.23 | | |
| FA12_HUMAN | 1.10E-01 | FMOD_HUMAN | 0.23 | | |
| APOE_HUMAN | 1.10E-01 | STT3A_HUMAN | 0.23 | | |
| FIBG_HUMAN | 1.12E-01 | HV102_HUMAN | 0.23 | | |
| PNPH_HUMAN | 1.13E-01 | LV147_HUMAN | 0.23 | | |
| CAVN3_HUMAN | 1.14E-01 | TGM2_HUMAN | 0.23 | | |
| CMA1_HUMAN | 1.14E-01 | THOC4_HUMAN | 0.24 | | |
| LUM_HUMAN | 1.15E-01 | TTHY_HUMAN | 0.25 | | |
| IGLL1_HUMAN | 1.15E-01 | BLVRB_HUMAN | 0.26 | | |
| KNG1_HUMAN | 1.17E-01 | C4BPA_HUMAN | 0.26 | | |
| A2MG_HUMAN | 1.17E-01 | OXSR1_HUMAN | 0.26 | | |
| HBA_HUMAN | 1.17E-01 | A1AG2_HUMAN | 0.26 | | |
| LG3BP_HUMAN | 1.23E-01 | IMA1_HUMAN | 0.27 | | |
| A1AT_HUMAN | 1.23E-01 | FABP5_HUMAN | 0.28 | | |
| FIBA_HUMAN | 1.25E-01 | IGHG2_HUMAN | 0.30 | | |
| CLUS_HUMAN | 1.27E-01 | CATG_HUMAN | 0.31 | | |

**Supplementary table 3.** A subset of prolactin inducible protein (PIP) spatial protein regulation adjacency values to other fully quantitated proteins (N=453) determined by adj_mod function of spatialHeatmap R package. The lower the adjacency value, the more inverse is the spatial regulation of the protein pair. In contrary, the values close to 1 show protein pairs obeying the same spatial regulation trends. The dark blue proteins (cutoff for adjacency value < 5.96e-04) showing inverse spatial regulation trends to PIP were subjected to STRING protein interaction and functional enrichment analysis. Keratins closely spatially regulated to PIP are highlighted by red color, while those with opposing regulatory trends are bolded.

| **Cellular_compartment_enrichment** | | | | | | |
|---|---|---|---|---|---|---|
| **term ID** | **term description** | **observed gene count** | **background gene count** | **strength** | **false discovery rate** | **matching proteins in the network (labels)** |
| **GOCC:0045111** | **Intermediate filament cytoskeleton** | 4 | 116 | 2.13 | **1.55E-05** | **KRT5,PLEC,KRT19,KRT18** |
| GOCC:0005882 | Intermediate filament | 3 | 73 | 2.21 | 0.00065 | KRT5,KRT19,KRT18 |
| GOCC:0099512 | Supramolecular fiber | 4 | 490 | 1.5 | 0.0015 | KRT5,PLEC,KRT19,KRT18 |
| GOCC:0043034 | Costamere | 2 | 13 | 2.78 | 0.0025 | PLEC,KRT19 |
| GOCC:0045095 | Keratin filament | 2 | 16 | 2.69 | 0.0031 | KRT5,KRT18 |
| GOCC:0005615 | Extracellular space | 4 | 985 | 1.2 | 0.009 | KRT5,PIP,KRT19,KRT18 |

| **Cellular_component_enrichment** | | | | | | |
|---|---|---|---|---|---|---|
| **term ID** | **term description** | **observed gene count** | **background gene count** | **strength** | **false discovery rate** | **matching proteins in the network (labels)** |
| **GO:0045111** | **Intermediate filament cytoskeleton** | 4 | 237 | 1.82 | **1.90E-04** | **KRT5,PLEC,KRT19,KRT18** |
| GO:0005882 | Intermediate filament | 3 | 199 | 1.77 | 0.0091 | KRT5,KRT19,KRT18 |
| GO:0043034 | Costamere | 2 | 19 | 2.61 | 0.0091 | PLEC,KRT19 |
| GO:0070062 | Extracellular exosome | 5 | 2099 | 0.97 | 0.0091 | KRT5,PIP,PLEC,KRT19, KRT18 |
| GO:0099512 | Supramolecular fiber | 4 | 939 | 1.22 | 0.0091 | KRT5,PLEC,KRT19, KRT18 |
| GO:0045095 | Keratin filament | 2 | 92 | 1.93 | 0.0349 | KRT5,KRT18 |

**Supplementary table 4** A detailed result of Cellular compartment and component enrichment analysis of a set of proteins displaying opposing spatial regulatory trends to prolactin inducible protein (PIP) in the STRING protein interaction and functional enrichment analysis [3]. Summed protein intensity for highlighted term (bold) was plotted in main text figure FIG6H.

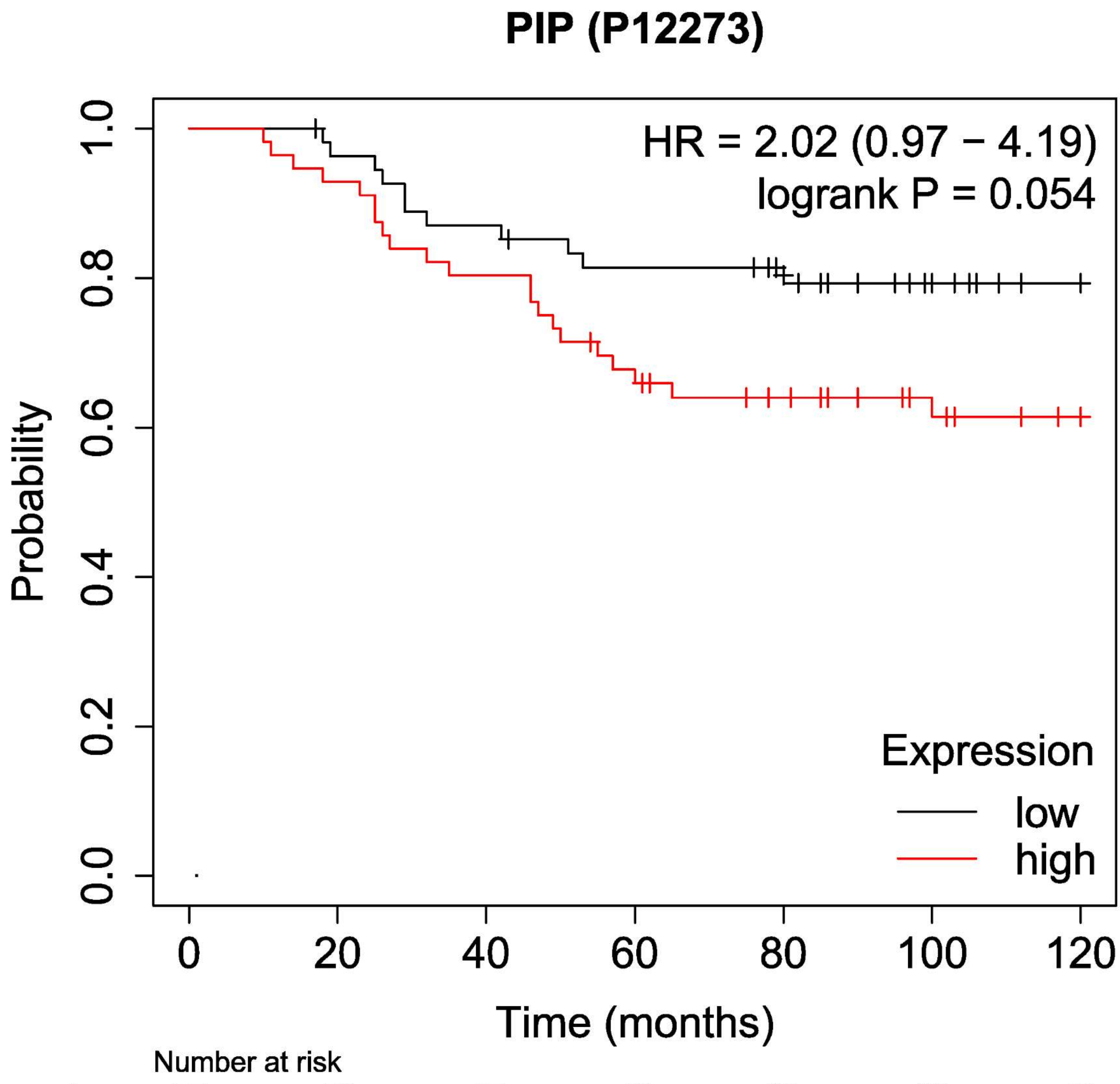


**Supplementary figure 6** A functional study of PIP relation to overall survival in breast cancer proteomic dataset. KMplot relates high and low expression of prolactin inducible protein (PIP) protein breast cancer (BC) patients (N=126) with overall survival in 120 months post diagnosis [4]. KMplot illustrates that high expression of PIP protein in BC patients leads to significantly lower overall survival (p = 0.054) if compared to overall survival in BC patients with low PIP expression within a timeline of 120 months post-diagnosis.

# SUPPLEMENTARY REFERENCES


1. Weke, K. *et al.* DIA-MS proteome analysis of formalin-fixed paraffin-embedded glioblastoma tissues. *Anal. Chim. Acta* **1204**, 339695 (2022).
2. Asleh, K. *et al.* Proteomic analysis of archival breast cancer clinical specimens identifies biological subtypes with distinct survival outcomes. *Nat. Commun.* **13**, 896 (2022).
3. Szklarczyk, D. *et al.* STRING v11: protein–protein association networks with increased coverage, supporting functional discovery in genome-wide experimental datasets. *Nucleic Acids Res.* **47**, D607–D613 (2019).
4. Ősz, Á., Lánczky, A. & Győrffy, B. Survival analysis in breast cancer using proteomic data from four independent datasets. *Sci. Rep.* **11**, 16787 (2021).
