# Supplementary material for: Novel FFPE proteomics method suggests prolactin induced protein as hormone induced cytoskeleton remodeling spatial biomarker
Source: Commun Biol. 2024 Jun 8;7:708. doi: 10.1038/s42003-024-06354-8 (PMC11162451; doi:10.1038/s42003-024-06354-8)
Supplement: Supplementary file 2 — Description of Additional Supplementary Materials [file 42003_2024_6354_MOESM2_ESM.docx]

**Description of Additional Supplementary Files**

**File name:** Supplementary Data 1

**Description:** Spatial protein regulation adjacency matrix of fully quantitated proteins (N=453) determined by the adj_mod function of the spatialHeatmap R package.

**File name:** Supplementary Data 2

**Description:** Complete numerical source data for graphs and charts in the manuscript and the supplementary information
