# Supplementary material for: Novel FFPE proteomics method suggests prolactin induced protein as hormone induced cytoskeleton remodeling spatial biomarker
Source: Commun Biol. 2024 Jun 8;7:708. doi: 10.1038/s42003-024-06354-8 (PMC11162451; doi:10.1038/s42003-024-06354-8)
Supplement: Supplementary file 5 — Reporting Summary [file 42003_2024_6354_MOESM5_ESM.pdf]

nature portfolio

Corresponding author(s): Dr. Jakub Faktor and Dr. Sachin Kote

Last updated by author(s): 2024/04/05

Reporting Summary

Nature Portfolio wishes to improve the reproducibility of the work that we publish. This form provides structure for consistency and transparency in reporting. For further information on Nature Portfolio policies, see our Editorial Policies and the Editorial Policy Checklist.

Statistics

For all statistical analyses, confirm that the following items are present in the figure legend, table legend, main text, or Methods section.

n/a	Confirmed	
☐	☒	The exact sample size (*n*) for each experimental group/condition, given as a discrete number and unit of measurement
☐	☒	A statement on whether measurements were taken from distinct samples or whether the same sample was measured repeatedly
☒	☐	The statistical test(s) used AND whether they are one- or two-sided<br>*Only common tests should be described solely by name; describe more complex techniques in the Methods section.*
☒	☐	A description of all covariates tested
☒	☐	A description of any assumptions or corrections, such as tests of normality and adjustment for multiple comparisons
☐	☒	A full description of the statistical parameters including central tendency (e.g. means) or other basic estimates (e.g. regression coefficient) AND variation (e.g. standard deviation) or associated estimates of uncertainty (e.g. confidence intervals)
☒	☐	For null hypothesis testing, the test statistic (e.g. *F*, *t*, *r*) with confidence intervals, effect sizes, degrees of freedom and *P* value noted<br>*Give P values as exact values whenever suitable.*
☒	☐	For Bayesian analysis, information on the choice of priors and Markov chain Monte Carlo settings
☒	☐	For hierarchical and complex designs, identification of the appropriate level for tests and full reporting of outcomes
☐	☒	Estimates of effect sizes (e.g. Cohen's *d*, Pearson's *r*), indicating how they were calculated

Our web collection on statistics for biologists contains articles on many of the points above.

Software and code

Policy information about availability of computer code

Data collection	Thermo Scientific Xcalibur 4.4.16.14 - data acquisition, acquisition method creation, operation of mass spectrometer and liquid chromatography system<br>FreeStyle 1.7 - RAW data visualization<br>Orbitrap Exploris480 Tune Application 4.2.362.16 - Mass spectrometer tuning and mass spectrometry data acquisition
Data analysis	1. Data conversion MSConvert version: 3.0.19094<br>2. DIA data extraction to pseudoDDA data: DIA-Umpire available in FragPipe (v.15)<br>3. Database search of MS/MS data: MSFragger 3.4 58 search engine embedded in FragPipe (v.15), Comet (Release 2020.01, revision 2) embedded in TPP v6.0.0. OmegaBlock 60 and MaxQuant 2.1.0.0 search engines<br>4. Posttranslational modification search: Crystal-C 64 and PTM-Shepherd, both implemented in FragPipe (v.15)<br>5. Peptide probability recalculation: PeptideProphet ProteinProphet as a part of the TPP v6.0.0. OmegaBlock or embedded in FragPipe (v.15).<br>6. Spectral library generation and DIA data extraction: Skyline-daily (64-bit, 20.1.9.234)<br>7. Statistical analysis of Skyline extracted DIA data and protein quantitation: R (version 4.0.0) package MSstats 4.0.1.<br>8. Spatial data analysis: spatiaHeatmap 2.3.0 R package running in R (version 4.0.0)<br>9. Plotting: Venn diagrams were generated in Eulerr 6.1.1 74 package. ggseqlogo 0.1 75 R package Ggpubr 0.4.0 package was used to stack multiple graphs per page. Circlize 0.4.15 76 was used to generate custom color palette for publication. Inkcape 1.2 and Gimp 2.10.32 were used to process the graphics to final panel plots and to generate svg image of breast FFPE tissue slide later used in spatialHeatmap R package. PCAtools 2.4.0 R package was used to plot PCA plot and heatmapply 1.4.2 R package was used to plot heatmaps.

For manuscripts utilizing custom algorithms or software that are central to the research but not yet described in published literature, software must be made available to editors and reviewers. We strongly encourage code deposition in a community repository (e.g. GitHub). See the Nature Portfolio guidelines for submitting code & software for further information.

Data

Policy information about availability of data

All manuscripts must include a data availability statement. This statement should provide the following information, where applicable:

- Accession codes, unique identifiers, or web links for publicly available datasets
- A description of any restrictions on data availability
- For clinical datasets or third party data, please ensure that the statement adheres to our policy

Mass spectrometry data including raw data files, search results and full protein quantitation results have been deposited to ProteomeXchange with dataset identifier: PXD037609 under username: reviewer pxd037609@ebi.ac.uk and password: fq17pAeG. All other data are available from the corresponding authors upon request.

Research involving human participants, their data, or biological material

Policy information about studies with human participants or human data. See also policy information about sex, gender (identity/presentation), and sexual orientation and race, ethnicity and racism.

Reporting on sex and gender	The study did not explicitly address statements about sex or gender. However, the studied material was derived from normal breast tissue retrieved during bilateral breast reduction mammoplasty predominantly involving individuals assigned female at birth. While the study material suggests a focus on the female sex, it is essential to acknowledge the absence of explicit statements regarding sex or gender in the research
Reporting on race, ethnicity, or other socially relevant groupings	The study did not explicitly address statements Reporting on race, ethnicity, or other socially relevant groupings. Given nature of study, which focuses mostly on methodological aspect of spatial proteomics of breast tissue FFPE slide from individual assigned female at birth, there is no applicable information to report regarding race, ethnicity, or other socially relevant groupings.
Population characteristics	This study does not include specific information on race, ethnicity, or other socially relevant groupings. The focus is rather methodological, particularly focused on spatial proteomics of any FFPE slide
Recruitment	The goal of the study is rather on methodological advancements in spatial proteomics using any FFPE slide. Nevertheless, using a single FFPE slide could evoke limitations or bias in participant recruitment. To address this we include in our study a benchmarking dataset (n= 10, FFPE breast normal tissue) processed classical way and an on-line available TMT breast cancer FFPE dataset, ensuring consistency in intensity-based protein rankings. This way we demonstrate biological significance of our result, by showing similar protein intensity ranking across studied datasets. This confirms that detected protein intensities represent rather genuine biologically relevant signals than contamination or bias.
Ethics oversight	Specimens were collected under clinical protocols approved by the Internal Review Board committee at Prince Hamza Hospital (No. 9/2019) and in accordance with ethical standards as laid down in the 1964 Declaration of Helsinki and its later amendments. Each participant involved underwent a careful and detailed informed consent process during their appointment. This informed and transparent process guaranteed that every participant had a clear understanding and willingly agreed to participate in the studies.

Note that full information on the approval of the study protocol must also be provided in the manuscript.

Field-specific reporting

Please select the one below that is the best fit for your research. If you are not sure, read the appropriate sections before making your selection.

☒ Life sciences ☐ Behavioural & social sciences ☐ Ecological, evolutionary & environmental sciences

For a reference copy of the document with all sections, see nature.com/documents/nr-reporting-summary-flat.pdf

Life sciences study design

All studies must disclose on these points even when the disclosure is negative.

Sample size	not applicable as spatial proteomics involved one FFPE slide cut into voxels
Data exclusions	FFPE Voxels number 2, 5, 8 were later suspended from the experiment due to suspicion to polymeric contamination originating from Parafilm seal that entered the insert during centrifugation and dipped into the extract.
Replication	Not applicable as spatial proteomics involved one FFPE slide cut into voxels. However, mass spectrometry intensity ranking of key proteins was screened in a benchmarking dataset (n = 11, FFPE breast normal tissue) processed classical way and in an on-line available TMT breast cancer FFPE dataset, ensuring consistency in intensity-based protein rankings in breast tissue. This way we demonstrate biological significance of our result, by showing similar protein intensity ranking across studied datasets. This confirms that detected protein intensities represent rather genuine biologically relevant signals than contamination or bias.

Randomization	not applicable as spatial proteomics involved on FFPE slide cut into voxels
Blinding	not applicable as spatial proteomics involved on FFPE slide cut into voxels

Reporting for specific materials, systems and methods

We require information from authors about some types of materials, experimental systems and methods used in many studies. Here, indicate whether each material, system or method listed is relevant to your study. If you are not sure if a list item applies to your research, read the appropriate section before selecting a response.

Materials & experimental systems

n/a	Involved in the study
☒	☐ Antibodies
☒	☐ Eukaryotic cell lines
☒	☐ Palaeontology and archaeology
☒	☐ Animals and other organisms
☒	☐ Clinical data
☒	☐ Dual use research of concern
☒	☐ Plants

Methods

n/a	Involved in the study
☒	☐ ChIP-seq
☒	☐ Flow cytometry
☒	☐ MRI-based neuroimaging

Plants

Seed stocks	not applicable
Novel plant genotypes	not applicable
Authentication	not applicable
